# Mapping the structural landscape of the yeast Ty3 retrotransposon RNA genome

**DOI:** 10.1093/nar/gkae494

**Published:** 2024-06-12

**Authors:** Angelika Andrzejewska-Romanowska, Julita Gumna, Ewa Tykwińska, Katarzyna Pachulska-Wieczorek

**Affiliations:** Department of RNA Structure and Function, Institute of Bioorganic Chemistry, Polish Academy of Sciences, Noskowskiego 12/14, 61-704 Poznan, Poland; Department of RNA Structure and Function, Institute of Bioorganic Chemistry, Polish Academy of Sciences, Noskowskiego 12/14, 61-704 Poznan, Poland; Department of RNA Structure and Function, Institute of Bioorganic Chemistry, Polish Academy of Sciences, Noskowskiego 12/14, 61-704 Poznan, Poland; Department of RNA Structure and Function, Institute of Bioorganic Chemistry, Polish Academy of Sciences, Noskowskiego 12/14, 61-704 Poznan, Poland

## Abstract

Long terminal repeat (LTR)-retrotransposons are significant contributors to the evolution and diversity of eukaryotic genomes. Their RNA genomes (gRNA) serve as a template for protein synthesis and reverse transcription to a DNA copy, which can integrate into the host genome. Here, we used the SHAPE-MaP strategy to explore Ty3 retrotransposon gRNA structure in yeast and under cell-free conditions. Our study reveals the structural dynamics of Ty3 gRNA and the well-folded core, formed independently of the cellular environment. Based on the detailed map of Ty3 gRNA structure, we characterized the structural context of *cis*-acting sequences involved in reverse transcription and frameshifting. We also identified a novel functional sequence as a potential initiator for Ty3 gRNA dimerization. Our data indicate that the dimer is maintained by direct interaction between short palindromic sequences at the 5′ ends of the two Ty3 gRNAs, resembling the model characteristic for other retroelements like HIV-1 and Ty1. This work points out a range of cell-dependent and -independent Ty3 gRNA structural changes that provide a solid background for studies on RNA structure-function relationships important for retroelement biology.

## Graphical Abstract

**Graphical Abstract:**
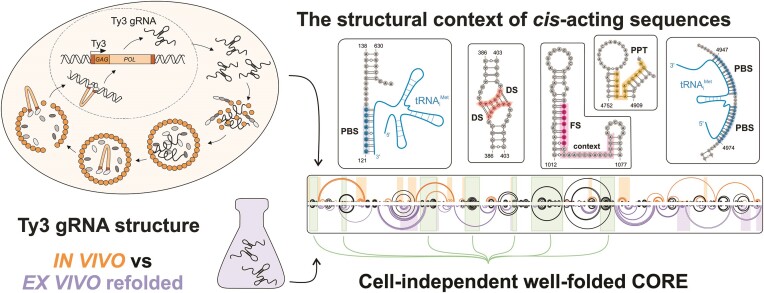

## Introduction

LTR-retrotransposons are endogenous reverse-transcribing single-stranded RNA viruses widely distributed in plants, fungi, and animals (1,2). Their RNA genome, akin to infectious RNA viruses, carries all essential instructions for replication and interactions with host cell machinery. In addition to genome function, it also serves as mRNA for element-encoded proteins. LTR-retrotransposons use a ‘copy-and-paste’ mechanism to propagate and spread within the host genome. New insertions of their copies lead to host genome remodeling and instability but may also be beneficial by providing regulatory sequences (3,4).

Among mobile genetic elements, the Ty3/*Gypsy* retrotransposons (*Metaviridae* family) are a prominent and evolutionarily important group, considered progenitors of retroviruses (5). Recently, their potential involvement in the process of sex chromosome differentiation was suggested (6). Also, one of the neuronal proteins, Arc, crucial for memory processes and synaptic plasticity, was indicated to be derived from Ty3/*Gypsy*, and like Ty3 Gag protein, can self-assemble into virus-like particles (VLPs) that encapsulate Arc mRNA (7,8). The biology of the Ty3/*Gypsy* class has been extensively studied. The Ty3 retrotransposon is their sole representative in the *Saccharomyces cerevisiae*. This element is one of the most characterized and broadly utilized models to understand how LTR-retroelements mobilize, interact with the cell machinery, and shape the host genome.

**Figure 1.**
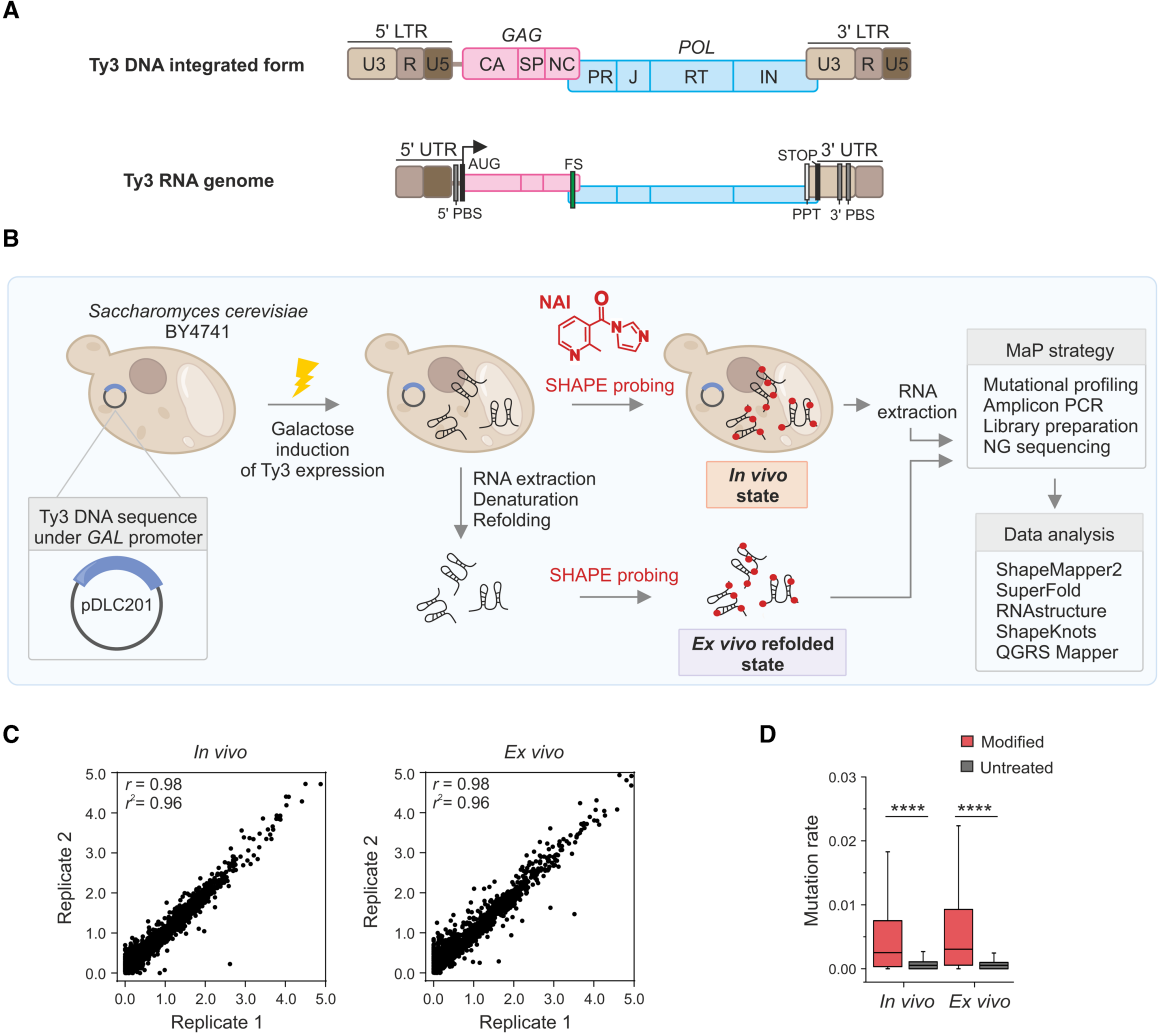
Ty3 DNA and gRNA organization, experimental strategy, and quality assessment of SHAPE-MaP data. (**A**) Organization of the Ty3 DNA and RNA genome. LTR – long terminal repeat, UTR - untranslated region, PBS - primer binding site, PPT - polypurine tract, FS – frameshift, CA - capsid domain, SP - short spacer, NC – nucleocapsid, PR – protease, J - junction domain, RT - reverse transcriptase, and IN - integrase. (**B**) Schematic presentation of the applied strategy. (**C**) Pearson correlation (*r*) plot of normalized SHAPE reactivities from two biological replicates for the *in vivo* and *ex vivo* SHAPE datasets, (*r*²) coefficient of determination. (**D**) Box plot analysis of mutation rates for modified and untreated samples from Ty3 gRNA probing under *in vivo* and *ex vivo* conditions. The boxes represent the interquartile range; a line indicates the median. Significance was computed by the Wilcoxon rank-sum test; ****P < 0.0001. The analysis for each nucleotide type separately is presented in Supplementary Figure S2.

The *S. cerevisiae* Ty3 gRNA is over 5 kb in length and contains two overlapping open reading frames (ORFs), *GAG* and *POL*, encoding structural and catalytic proteins, flanked by 5′ and 3′UTRs (193 and 214 nts, respectively) (5) (Figure 1A). The first step of Ty3 replication involves element transcription from the DNA copy integrated with the host genome. After export from the nucleus to the cytoplasm, Ty3 gRNA is utilized as mRNA for translation of Gag and Gag-Pol or is directed to cytoplasmic foci, termed retrosomes (9,10), where a full-length Ty3 gRNA and proteins assemble into VLPs. Finally, mature VLPs contain Gag-derived structural proteins, enzymes encoded by *POL*, at least two copies of Ty3 gRNA in the dimeric form, and some cellular factors (e.g. tRNA_i_^Met^) (11–13). Ty3 capsid morphology and structure display striking similarities to mature HIV-1 capsids (14). In mature VLPs, gRNA is reverse transcribed to cDNA using self-encoded reverse transcriptase and cellular tRNA_i_^Met^ as a primer. The final step of the replication includes integrating Ty3 DNA copy into the transcription start site of genes transcribed by RNA polymerase III (15).

Analogous to retroviruses, the 5′ and 3′ termini of Ty3 gRNA are rich in regulatory sequences directly involved in replication. The minimal Ty3 element capable of retrotransposition, when Ty3 proteins are provided in *trans*, encompasses the entire 5′ UTR and part of both *GAG* ORF and 3′ UTR (miniTy3: +1–426 nt and + 4980–5052 nt) (16). Ty3 contains a bipartite primer binding site (PBS) with the 5′ and 3′ portions located at opposite ends of the gRNA (17) (Figure 1A). This specific tRNA_i_^Met^ binding is proposed to mediate gRNA cyclization and dimerization. Replication of Ty3 requires direct interaction between retrotransposon gRNA and Gag3 (18). Moreover, it was shown *in vitro* that the nucleic acid chaperone activity of Gag3-derived nucleocapsid protein (NCp9) facilitates tRNA_i_^Met^ annealing, cyclization and Ty3 gRNA dimerization via tRNA_i_^Met^–tRNA_i_^Met^ bridge (17,19). However, the *in vivo* studies do not support PBS role in dimerization and packaging of Ty3 gRNA (13,20). *Cis*-acting sequences essential for Ty3 gRNA localization in retrosomes and packaging to VLPs are not precisely defined, but both UTRs and *POL* are needed for the proper functioning of these processes (20,21).

Recent studies have highlighted the importance of investigating the relationship between RNA structure and function to better understand RNA-mediated cellular processes and viral RNA acting (22,23). Hence, the knowledge of the native structure of retrotransposon RNA genomes exposed to the cellular environment is necessary to understand comprehensively how genome architecture influences the retrotransposition process. However, there is still very little known about the folding of retrotransposon RNA genomes, and the only gRNA *in vivo* structure model is currently available for yeast Ty1 LTR-retrotransposon (*Pseudoviridae* family) (24,25). Until now, the structure of the Ty3 RNA genome remains unknown.

Here, we report the complete secondary structure model of the Ty3 retrotransposon RNA genome in living cells and under cell-free conditions. Using the SHAPE-MaP (Selective 2′-Hydroxyl Acylation analyzed by Primer Extension and Mutational Profiling) strategy, we characterized the state-specific structural folding of the retrotransposon gRNA. In Ty3 gRNA, we have identified a well-folded core that forms independently from the cellular environment and encompasses almost 20% of the sequence. We show the structural context of the known Ty3 *cis*-acting primer binding site (PBS), polypurine tract (PPT), and frameshift (FS) sequence. We also point out stable gRNA motifs, which may possess a functional role during retrotransposition. Finally, we identify a novel functional sequence critical for Ty3 gRNA dimer formation *in vitro*—dimerization site (DS). We also characterize gRNA key structural features shared between representative retroelements.

## Materials and methods

### Yeast strain and growth conditions

*S. cerevisiae* strain BY4741 (*MATa his3Δ1 leu2Δ0 met15Δ0 ura3Δ0*) (Horizon Discovery) were transformed with pDLC201 (Addgene plasmid #113388; formerly pEGTy3-1, 2μ, URA3, galactose inducible Ty3-1) (11). Yeast transformation was performed by the lithium acetate procedure (26,27). *Escherichia coli* strain DH5α (F^–^ φ80*lac*ZΔM15 Δ(*lac*ZYA-*arg*F)U169 *rec*A1 *end*A1 *hsd*R17(r_K_^–^, m_K_^+^) *pho*A *sup*E44 λ^–^*thi*-1 *gyr*A96 *rel*A1) (Invitrogen) was used for DNA cloning and plasmid preparation. *E. coli* cells were grown in LB broth + 0.1 mg/ml Ampicillin (or plates). For non-selective growth, yeast cells were grown on a YPDA medium (BioShop). Plasmid selection used a synthetic yeast growth medium with a drop-out mix (SC-Ura). The presence of plasmid in the transformed yeast strain was confirmed by colony PCR. Used primer sequences are listed in Supplementary Table S1.

For SHAPE-MaP experiments, BY4741 containing pDLC201 plasmid was grown in SC-Ura 2% raffinose broth at 30°C with constant shaking at 200 rpm. Saturated cultures were diluted to OD_600 nm_ of approximately 0.1 with SC-Ura 2% galactose broth to induce Ty3 expression from the *GAL* promoter. Cultures were grown to a final OD_600 nm_ of 0.7–0.8 at 30°C with constant shaking at 200 rpm.

### DNA, RNA and protein substrates

The template for transcription of unmodified yeast tRNA_i_^Met^ was generated by PCR, and RNA was synthesized using MEGAshortscript T7 Transcription Kit (Invitrogen). RNA was purified by denaturing gel electrophoresis (8 M urea) in 1× TBE buffer, eluted from the gel matrix, and concentrated by ethanol precipitation. The DNA templates for *in vitro* transcription of Ty3 5′ RNA (+1–429 nt), 3′ RNA (+4624–5052 nt), and ΔPAL6 5′ RNA (+1–390 nt) were obtained by PCR amplification from plasmid pDLC201. All primers are listed in Supplementary Table S1. Transcripts were synthesized using MAXIscript T7 Transcription Kit (Invitrogen) according to the manufacturer's protocols and purified using a Monarch RNA Cleanup Kit (New England BioLabs). Transcripts integrity was monitored by agarose gel electrophoresis under denaturing conditions. RNA 3′-end labeling with fluorescent dye was carried out overnight at 4°C in an 18 μL reaction containing 20U of T4 RNA Ligase (Thermo Fisher Scientific Inc.), 1× T4 RNA Ligase Buffer, 20 μM ATP, 20 μM pCp-Cy5 or pCp-Cy3 (Jena Bioscience), and 30 pmol of RNA. Labeled RNA was purified using a Monarch RNA Cleanup Kit (New England BioLabs). Purified transcripts were stored at –20°C.

The Ty3 NCp9 protein (57 amino acids: TVRTRRSYNKPMSNHRNRRNNNPSREECIKNRLCFYCKKEGHRLNECRARKASSNRS) was prepared by chemical synthesis and purified by high-performance liquid chromatography (HPLC) (GenScript). NCp9 stocks were reconstituted at 0.8 mg/ml in a buffer containing 20 mM HEPES pH 6.5, 30 mM NaCl, 5 mM dithiothreitol, 0.15 mM ZnCl_2_ and 10% glycerol.

### Ty3 RNA dimerization and tRNA_i_^Met^ annealing assays

Cy3-labeled Ty3 RNA (0.5 pmol) was refolded in buffer containing 40 mM Tris–HCl pH 8.0 and 130 mM KCl by heating at 95°C for 3 minutes, slowly cooled to 60°C, placed on ice for 2 minutes, and then incubated at 37°C for 30 min following the addition of MgCl_2_ to 4 mM. For assays in the presence of tRNA_i_^Met^, Cy5-labeled tRNA was folded separately in equivalent conditions. Ty3 RNA was combined with tRNA_i_^Met^ at a 1:1 molar ratio before adding NCp9. RNAs were incubated with increasing protein concentrations at 37°C for 30 min. The reactions were stopped by incubation with a quenching solution (1% SDS, 5 mM EDTA) at room temperature for 5 min. The samples were phenol/chloroform extracted, and 15 μl of aqueous phase was mixed with 3 μl of 25% Ficoll 400. RNA was resolved on a 1.3% agarose gel in 0.5× TB at room temperature. The gels were quantified by imaging using Amersham Typhoon 5 Biomolecular Imager with ImageQuantTL v10.1 software (Cytiva). The obtained data were analyzed using GraphPad Prism 8 (GraphPad Software). In all cases, at least four independent experiments were performed, and the data presented are representative of the whole. The reproducibility of the experiments was assessed by standard deviation.

### *In vivo* SHAPE modification

A 150 ml culture of exponentially growing yeast cells was centrifuged, and the cell pellet was washed once with cold PBS and then resuspended in the desired amount of PBS. Each sample was divided into two equal amounts, and 1 M NAI in DMSO (Merck) was added at a final concentration of 100 mM. A corresponding amount of DMSO was added to a control sample. The modification reactions were conducted at 30°C for 20 min, followed by quenching of NAI by adding DTT at a final concentration of 500 mM. Cells were collected at 8000 × g (10°C) for 5 min, followed by total RNA extraction.

For both experimental states (*in vivo* and *ex vivo* refolded states), different NAI concentrations and modification times were tested, and conditions yielding the efficient NAI-induced mutation rate were chosen for final experiments.

### Extraction of total RNA

RNA isolation was performed as previously described (24). Briefly, yeast pellets were resuspended in 1 ml of lysis buffer (10 mM Tris–HCl, pH 8.5, 5 mM EDTA, 2% SDS, 2% 2-mercaptoethanol) and incubated at 83°C for 20 min with constant shaking at 450 rpm. The tubes were centrifuged at 12 000 × g for 5 min. The supernatants were transferred to fresh tubes, and RNA was extracted twice with phenol (pH 8.0), followed by two extractions with phenol : chloroform (pH 4.5). RNA was recovered by LiCl precipitation overnight at –20°C. RNA pellets were washed twice in 70% ethanol and resuspended in 30 μl of water. After *in vivo* SHAPE modification, samples were treated with DNase I (Ambion) and purified on an affinity column (RNeasy Mini Kit; QIAGEN). The integrity of the total RNA was evaluated using Qubit™ RNA IQ Assay (Invitrogen), and RNA IQ numbers were >8.5 for all samples. Purified RNA samples were stored at –20°C.

### *Ex vivo* RNA folding and SHAPE modification

For RNA folding, ∼40 μg of total RNA in a volume of 120 μl was denatured at 95°C for 2 min, then transferred immediately to ice and incubated for 2 min. Then, 60 μl of 3.3 x RNA folding buffer (333 mM HEPES, pH 8.0; 333 mM NaCl; 20 mM MgCl_2_) was added. RNA was then incubated for 20 min at 30°C to allow secondary structure formation. For *ex vivo* SHAPE modification, each sample of refolded RNA was divided into two equal amounts, and 1 M NAI in DMSO was added at a final concentration of 100 mM. A corresponding amount of DMSO was added to a control sample. The modification reactions were carried at 30°C for 15 min, followed by quenching with DTT. RNA was recovered by ethanol precipitation overnight at -20°C. RNA pellets were washed twice in 70% ethanol and resuspended in 30 μl of water. Samples were then treated with DNase I (Ambion) and purified on an affinity column (RNeasy Mini Kit; QIAGEN).

### *In vitro* SHAPE modification

Ty3 RNA (3 pmol) in the presence or absence of tRNA_i_^Met^ (3 pmol) was folded as described for dimerization/annealing assays. Subsequently, 600 pmol of NCp9 or an equal volume of protein buffer were added to the RNA mixture. The reaction was incubated at 37°C for 30 min and stopped by incubation with a quenching solution at room temperature for 5 min, followed by phenol/chloroform extraction. Each reaction was divided into two tubes and treated with NAI in DMSO at a final concentration of 100 mM or DMSO alone. The modification reactions were carried out for 15 min at 24°C, due to Ty3 5′ RNA dimer instability at 30°C after protein removal (Supplementary Figure S4). The same modification conditions were used for Ty3 3′ RNA. SHAPE modification was followed by quenching with DTT. To verify the efficiency of the reaction, one-third of the RNA was resolved on a 1.3% agarose gel in 0.5× TB. The rest of the RNA was recovered by ethanol precipitation overnight at –20°C. RNA pellets were washed twice in 70% ethanol and resuspended in 30 μl of water. Detection of 2′-*O*-adducts and data processing were performed as described below. The contribution of RNA dimer to the ensemble SHAPE reactivity profile was calculated as previously described (28,29). Briefly, the SHAPE reactivity value of each nucleotide obtained for the mixture of RNA monomer and dimer is a sum of reactivity values of both states, weighted according to the fractional contribution of each to the total RNA population.

### Amplicon SHAPE-MaP

To apply the SHAPE-MaP strategy to the Ty3 gRNA under *in vivo* and *ex vivo* states, eight pairs of primers for overlapping amplicons, tiling the entire genome length, were designed. For *in vitro* experiments, two additional shortened amplicons for 5′ and 3′ ends were also designed. Amplicon-specific reverse transcription was performed as described previously (30). In brief, 1 ug of total RNA or 1 pmol of *in vitro* transcribed RNA was mixed with 1 μl of the corresponding 2 μM reverse primer. Primers were annealed at 65°C for 5 min and then cooled to 4°C, followed by the addition of 8 μl of 2.5× MaP buffer (125 mM Tris, pH 8.0; 187.5 mM KCl; 15 mM MnCl_2_; 25 mM DTT and 1.25 mM dNTPs) and incubation at 42°C for 2 min. After the addition of 1 μl of SuperScript II reverse transcriptase (Invitrogen), the reaction in 20 μl of total was incubated at 42°C for 3 h, and then RT was heat-inactivated at 70°C for 15 min. The generated cDNA was purified using ZR DNA Sequencing Clean-Up Kit (Zymo Research). 2 μl 2 M NaOH was added to each reaction and incubated at 95°C for 5 min to degrade the RNA, followed by cooling on ice and the addition of 2 μl 2 M HCl to neutralize the reaction. cDNA was again purified using the ZR DNA Sequencing Clean-Up Kit. dsDNA amplicons, tilling the Ty3 genome, were generated using NEBNext Ultra II Q5 Master Mix (New England BioLabs), amplicon-specific forward and reverse primers, and 1/10 of purified cDNA. PCR products were visualized on 1.2% agarose gel to confirm the production of correct-sized amplicons, and the desired amplicon products were extracted using Monarch DNA Gel Extraction Kit (New England BioLabs). Accurate concentration of dsDNA amplicons was measured by Qubit dsDNA High Sensitivity assay (Thermo Fisher Scientific Inc.). All primers are listed in Supplementary Table S1.

### Illumina sequencing

The dsDNA amplicons were equimolarly pooled to yield the final odd and even sets. Obtained pools were fragmented and proceeded toward downstream library preparation by Genomed (Poland) or Novogene (UK). Sequencing was performed on Illumina MiSeq and NovaSeq instruments, outputting 2 × 300 or 2 × 250 paired-end data sets.

### MaP analysis

Read quality assessment was performed using the FastQC (https://www.bioinformatics.babraham.ac.uk/projects/fastqc/). All SHAPE-MaP data were analyzed using the ShapeMapper 2 pipeline (42) and aligned to the sequence of the Ty3 RNA genome (YGRWTy3-1, SGD database). Due to repeated sequence (R) at both ends of the Ty3 genome, odd and even libraries were analyzed separately. The read-depth threshold setting of 5000× was used as a quality control benchmark. All libraries passed the three quality-control checks of ShapeMapper 2.

### RNA structure modeling

To Ty3 RNA genome MFE structure prediction, pairing probabilities calculation and lowSS regions identification, the SuperFold software (30) using functions implemented in RNAstructure (31) was applied. Obtained SHAPE reactivities for replicates were averaged and used as pseudo-energy constraints. A default value of maximum pairing distance of 600 nt was imposed to force the prediction of local base pairs. Slope and intercept folding parameters were set to 1.8 and –0.6 kcal mol^–1^, respectively. RNA structures were visualized with StructureEditor, a tool in the RNAstructure package, and VARNA (31,32).

To *de novo* identify pseudoknots in the *in vivo* and *ex vivo* Ty3 genome, the ShapeKnots software with experimental SHAPE constraints was used (33). Folding was performed in a 600-nt sliding window with 100-nt increments, which minimized biases resulting from sequence shortening and enabled the prediction of multiple pseudoknots in one RNA molecule. Two additional folds in different window sizes (800-nt and 1000-nt) were computed at the 5′ and 3′ ends to increase the sampling of terminal regions. To *in silico* prediction of potential RNA G-quadruplexes, QGRS Mapper was applied (34).

### Sensitivity and positive predictive value (PPV)

The sensitivity and PPV for the obtained MFE models were calculated using the Scorer function (implemented in RNAstructure). Sensitivity defines the percentage of base pairs in the reference MFE structure that are also present in the compared MFE structure, and the PPV describes the percentage of base pairs in the MFE structure that are present in the reference model. If the region contained only part of the predicted helix, calculations were performed with the manual addition of nucleotides from the disrupted helix.

### Gini index

The Gini index was calculated using the R package (R Core Team, 2021). Based on the assessment of SHAPE reactivity profile inequality, this metric defines the level of RNA structure. A relatively even distribution of SHAPE modifications is reflected by a Gini index close to zero, and occurs when RNA is unfolded or RNA structure is highly heterogeneous. A Gini index close to one occurs when a subset of residues is strongly protected from SHAPE, and indicates a highly stable RNA structure.

### Local MFE and HP bps content

Local bps content was computed by calculating the percentage of nucleotides engaged in MFE or HP base pairs within a 75-nt sliding window across the Ty3 genome (in steps of 1 nt). Local bps content values were plotted in a heatmap using the R package (R Core Team, 2021).

### Statistical analysis

Statistical analysis of SHAPE-MaP data, including Pearson's correlation, Student's *t*-test, and Wilcoxon rank sum test, was computed using statistical functions in Microsoft Excel and the R package (R Core Team, 2021). The ΔSHAPE framework was applied to detect statistically significant RNA structural differences between states (35). Data visualization was performed using the OriginLab and R package.

## Results

### *In vivo* and *ex vivo* mapping of the Ty3 gRNA secondary structure

To explore the secondary structure of the Ty3 retrotransposon gRNA, we utilized a high-throughput SHAPE-MaP strategy (36) (Figure 1B). In general, SHAPE uses small electrophilic reagents to measure the conformation of each nucleotide by forming covalent adducts at the flexible RNA ribose 2′-OH group (37). Thus, high SHAPE reactivities (a high level of SHAPE modification) indicate flexible or unpaired nucleotides, while low SHAPE reactivities point out structurally constrained or base-paired nucleotides. To probe RNA molecules, we used NAI (2-methylnicotinic acid imidazolide), the SHAPE reagent known to effectively penetrate the cell wall and membranes (38). In the applied strategy, modifications are read out by mutational profiling (MaP). Under appropriate experimental conditions, the modified nucleotides induce the mutations in the cDNA sequence during reverse transcription rather than a stop. Combining this experimental approach with NGS allows the readout of multiple modifications from a single sequencing read, making the method very sensitive and accurate in determining the structure of RNA and enabling the study of the secondary structure, even of rare or less stable RNAs (30).

The reference *S. cerevisiae* strain BY4741 genome contains only two full-length Ty3 elements, one of which, YGRWTy3-1, is transpositionally active (5,11). However, multiple incomplete Ty3 sequences, including solo LTRs, are also present. In nature, transcription and replication of the Ty3 are induced by pheromone stimulation in mating yeast, but retrotransposition is a relatively rare event (39). To obtain structural data for an active and more homogenous pool of Ty3, we used the yeast strain BY4741 with Ty3-1 expressed from the galactose-inducible promoter on a high-copy plasmid (Figure 1B). This system enables synchronous Ty3 expression and retrotransposition regardless of the cell cycle (11). Ty3 gRNA participates in all replication steps, and its major pool is present in the cytoplasm or retrosomes, while about 25% is packaged to VLPs (40,41). To determine the impact of cell environment on Ty3 gRNA architecture, we performed RNA structure mapping in living cells (*in vivo* state) or after extraction and refolding of total yeast RNA (refolded *ex vivo* state). Ty3 gRNA isolated from cells is devoid of all protein factors that can impact RNA folding but bears the same post-transcriptional RNA modifications as probed under *in vivo* conditions.

To analyze the complete retrotransposon gRNA, which spans 5052 nucleotide residues, we created eight overlapping amplicons, each covering approximately 730 nucleotides of the Ty3 gRNA sequence. During the optimization step, various NAI concentrations and SHAPE reaction times were tested (Supplementary Figure S1). We produced several biological and technical replicates, resulting in highly reproducible data. Finally, two independent biological replicates of SHAPE-MaP experiments in yeast and under *ex vivo* conditions were conducted using NAI at a final concentration of 100 mM for 20 and 15 min, respectively. All data were analyzed using the ShapeMapper2 pipeline (42). The resulting mutation profiles indicated a strong correlation between the replicates (*in vivo* replicates *r* = 0.98; *ex vivo* replicates *r* = 0.98; Figure 1C). Importantly, we observed significantly elevated mutation rates in NAI-treated samples, compared to untreated samples, proving a high efficiency of SHAPE modification in both experimental states (*P* < 0.0001; Figures 1D and Supplementary Figure S2). Finally, the SHAPE-MaP data with a median effective depth > 50 000 reads per site in each replicate provided effective reactivity information for 99.1% of nucleotides in the Ty3 gRNA. That guaranteed the correctness of our strategy, and we used these datasets for further analyses in this work.

### Model-free analysis of the structural propensity in the Ty3 RNA genome

**Figure 2.**
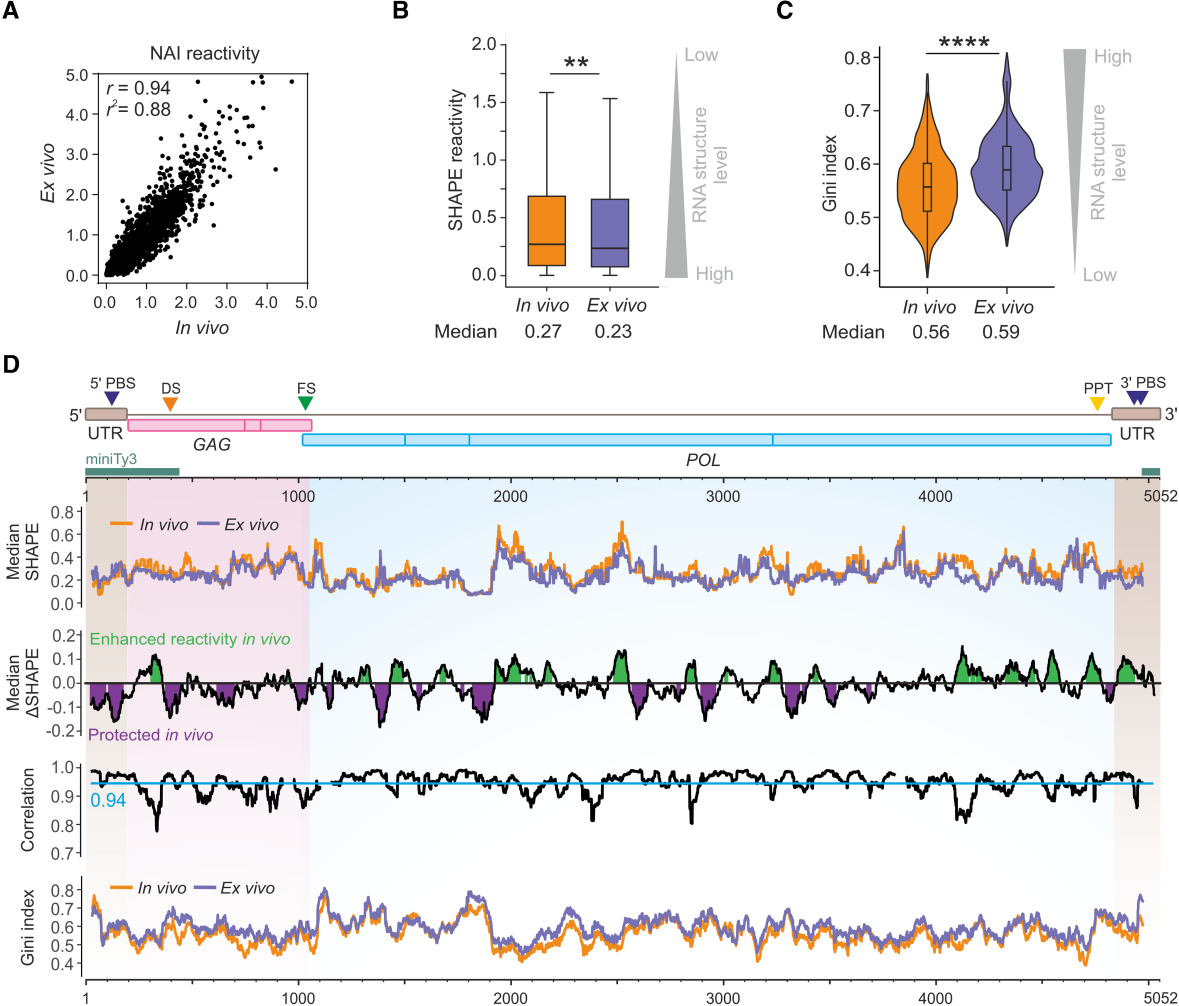
SHAPE-based comparison between *in vivo* and *ex vivo* structure of the Ty3 gRNA. (**A**)> Pearson correlation (*r*) plot of normalized SHAPE reactivities between *in vivo* and *ex vivo* datasets. (**B**) Box plot analysis of SHAPE reactivity distributions with medians. (**C**) Violin plot analysis of Gini index distributions calculated in a 51-nt sliding window with medians. Significance was computed by the Wilcoxon rank-sum test; ****P < 0.0001; **P < 0.01. (**D**) Profiles of the median SHAPE (upper plot), ΔSHAPE (*in vivo – ex vivo*, upper middle plot), Pearson correlation (lower middle plot), and Gini indexes distributions (lower plot), smoothed with a 51-nt sliding window. A more detailed presentation of ΔSHAPE analysis was presented in Supplementary Figure S3. The *cis*-acting sequences are indicated. The dimerization site (DS) was identified in this work. Localization of miniTy3 sequences was marked as dark green boxes. The nucleotide numbering in the text and all figures refers to the Ty3 gRNA sequence.

First, we conducted the model-free analysis of the SHAPE-MaP data obtained for Ty3 gRNA. The *in vivo* and *ex vivo* SHAPE reactivity datasets exhibited a very strong global correlation (*r* = 0.94, Figure 2A). Concurrently, the global median SHAPE reactivity was higher in the cell (Figure 2B), suggesting a slightly more unfolded RNA structure. As single-value results may not accurately reflect local characteristics of long RNA, we used a sliding window algorithm to capture unique structural features of Ty3 gRNA states. Based on the analysis of median SHAPE and ΔSHAPE (*in vivo* – *ex vivo*) reactivity profiles, we identified statistically important differences in many gRNA regions (Figure 2D and Supplementary Figure S3) (35). These differences were evident independently of the applied window size (Supplementary Figure S3). Also, the correlation profile dropped below the global value in many regions, indicating sites of decreased structural similarity (Figure 2D). Furthermore, the distribution of Gini indexes—a metric used to measure inequality - had higher *ex vivo* values than the *in vivo* state (median Gini - *in vivo*: 0.56; *ex vivo*: 0.59; Figure 2C and D). This additionally implied a lower level of the Ty3 gRNA structural organization *in vivo*. Together, model-free analyses have revealed both environment-specific features and regions of exceptionally high similarity within Ty3 gRNA.

### SHAPE-directed structure prediction revealed a well-folded core of Ty3 gRNA

**Figure 3.**
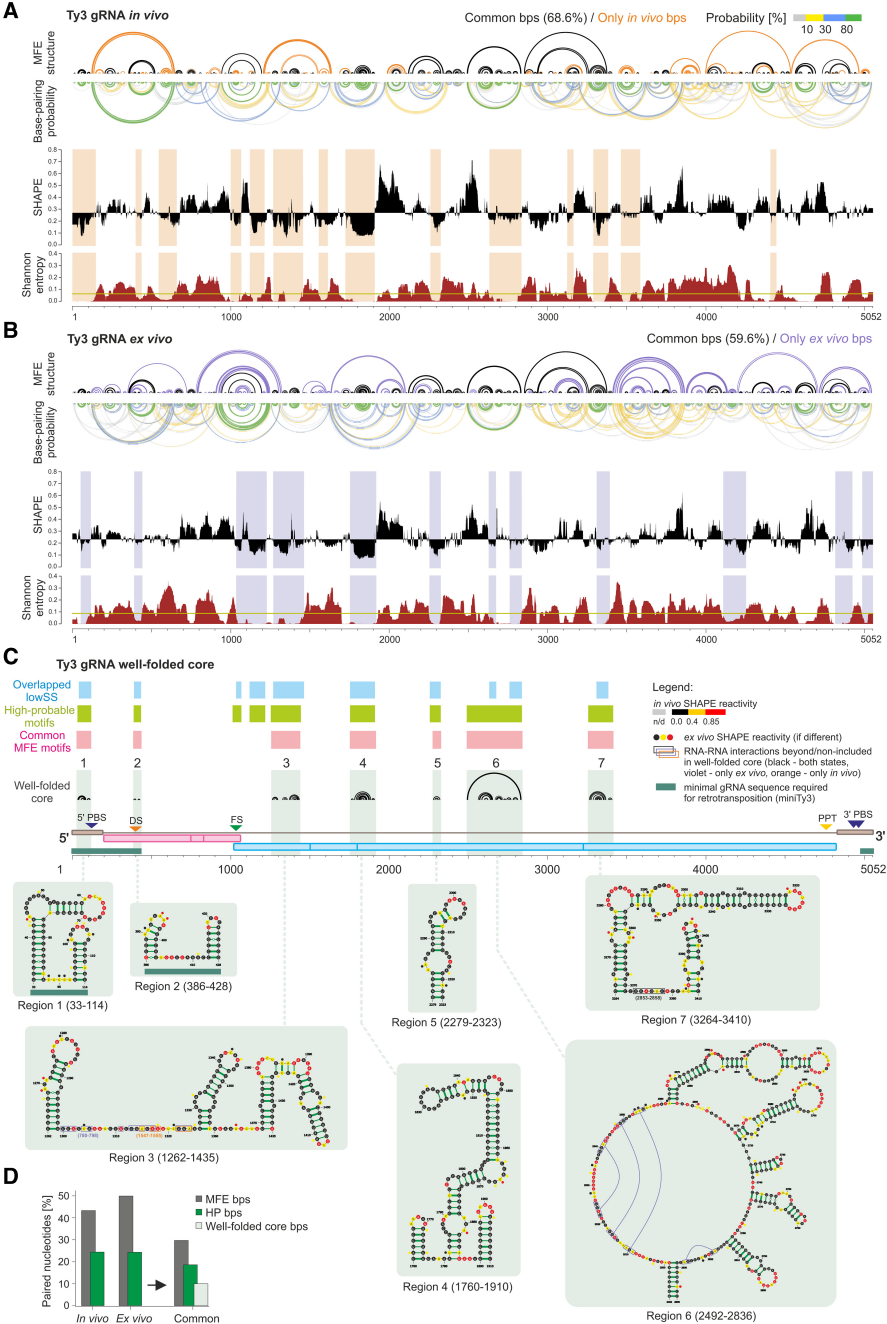
SHAPE – based prediction of the Ty3 gRNA structure and well-folded core. (**A**)> Structure map of the Ty3 gRNA *in vivo* depicting: arc plots of predicted MFE structure and base-pairing probabilities (see scale); median SHAPE reactivity profile with respect to the global median reactivity and Shannon entropy profile (both in 51-nt sliding window). Shadings mark lowSS regions. (**B**) shows the analogous analysis performed for *ex vivo* Ty3 gRNA. (**C**) Well-folded core identification. Black arcs represent the MFE structure of the core regions. Detailed secondary structure models are presented below. Nucleotides are colored by SHAPE reactivity (see scale), HP bps are marked in green. (**D**) Percentages of paired nucleotides in the predicted MFE structures, including HP bps and well-folded core.

Next, using obtained SHAPE-MaP data as constraints, we modeled the secondary structure of the Ty3 retrotransposon gRNA with the SuperFold pipeline (Figure 3) (30). The consensus minimum-free energy (MFE) secondary structure model of Ty3 gRNA predicted with *in vivo* data contained slightly fewer base-paired nucleotides, lower GC bp content, and accordingly, exhibited a weaker folding strength, than the *ex vivo* model (Table 1 and Figure 3A, B, and D). The positive predictive value (PPV) and the sensitivity—metrics of RNA structure similarity—were 68.6% and 59.6%, respectively (Figure 3A and B, black arcs). Consequently, the predicted MFE structure models differed more than the global SHAPE reactivity correlation suggested. These comparisons indicate that minor and local SHAPE reactivity changes, which did not impact correlation value, can significantly affect RNA structure prediction.

**Table 1.** Δ*G* energy and base pair content calculations for predicted RNA MFE models and RNA–RNA interactions

	Δ*G* energy (kcal/mol)	Total bp	GC bp
**RNA**			
Ty3 gRNA *in vivo*	–769.2	1095	481
Ty3 gRNA *ex vivo*	–844.3	1260	546
tRNA_i_^Met^	–33.9	21	18
**RNA–RNA interactions**			
Ty3 5′ PBS-tRNA_i_^Met^	–12.2	8	5
Ty3 3′ PBS-tRNA_i_^Met^ (part 1)	–14.8	12	6
Ty3 3′ PBS-tRNA_i_^Met^ (part 2)	–20.5	11	8

To account for the structural dynamics of the Ty3 gRNA in both states, we conducted a thorough analysis of the probability of forming each base pair. This approach allowed us to characterize the structural heterogeneity across the RNA molecule and differentiate between RNA regions with non-mutually exclusive base pairs that fold into one stable conformation versus highly dynamic regions. Our calculations revealed that in both states, a comparable number of nucleotides (∼24%) were involved in highly probable base pairs (HP bps, defined as having over 80% probability) (Figure 3A, B and D). Over 76% of the predicted HP bps were common for the *in vivo* and *ex vivo* Ty3 gRNA states. These results show that there is a significant group of HP bps that are formed regardless of the RNA folding environment. Consistent with the model-free analysis, in the predicted MFE structure, the 5′ region of Ty3 gRNA is much more stabilized *in vivo* than *ex vivo* (higher content of HP bps, low level of alternative pairings) (Figure 3A).

To search for more stable and structured regions, we identified RNA positions with both low SHAPE reactivity and low Shannon entropy (lowSS regions) (30). For Ty3 gRNA, we identified 14 and 12 well-folded regions under *in vivo* and *ex vivo* states, respectively (Figure 3A and B, light orange and violet shadings). The 1st and 2nd lowSS regions were contained within the miniTy3 sequence (16). We found that most of the functional *cis*-acting sequences of Ty3, including the 5′ PBS (17), FS (43) and DS (this work) were localized within lowSS regions identified *in vivo*. Furthermore, we dissected 9 lowSS regions that overlapped between states. Among them, seven shared the same secondary structure motifs enriched in the HP bps (Figure 3C). Therefore, we propose that these lowSS regions form a state-independent well-folded gRNA core. The core covered about 19.4% of the Ty3 gRNA sequence and included the most common HP base pairs (Figure 3D). Much of the core sequence accumulated within or in the vicinity of the region coding reverse transcriptase, which is known to be the most conserved domain among retroelements (44). Even outside of the identified well-folded core, the RT-coding region exhibits a very high level of structural similarity across the states (PPV – 90.5%, sensitivity – 82.8%).

### *In vitro* analysis of RNA–RNA interactions essential for Ty3 replication

**Figure 4.**
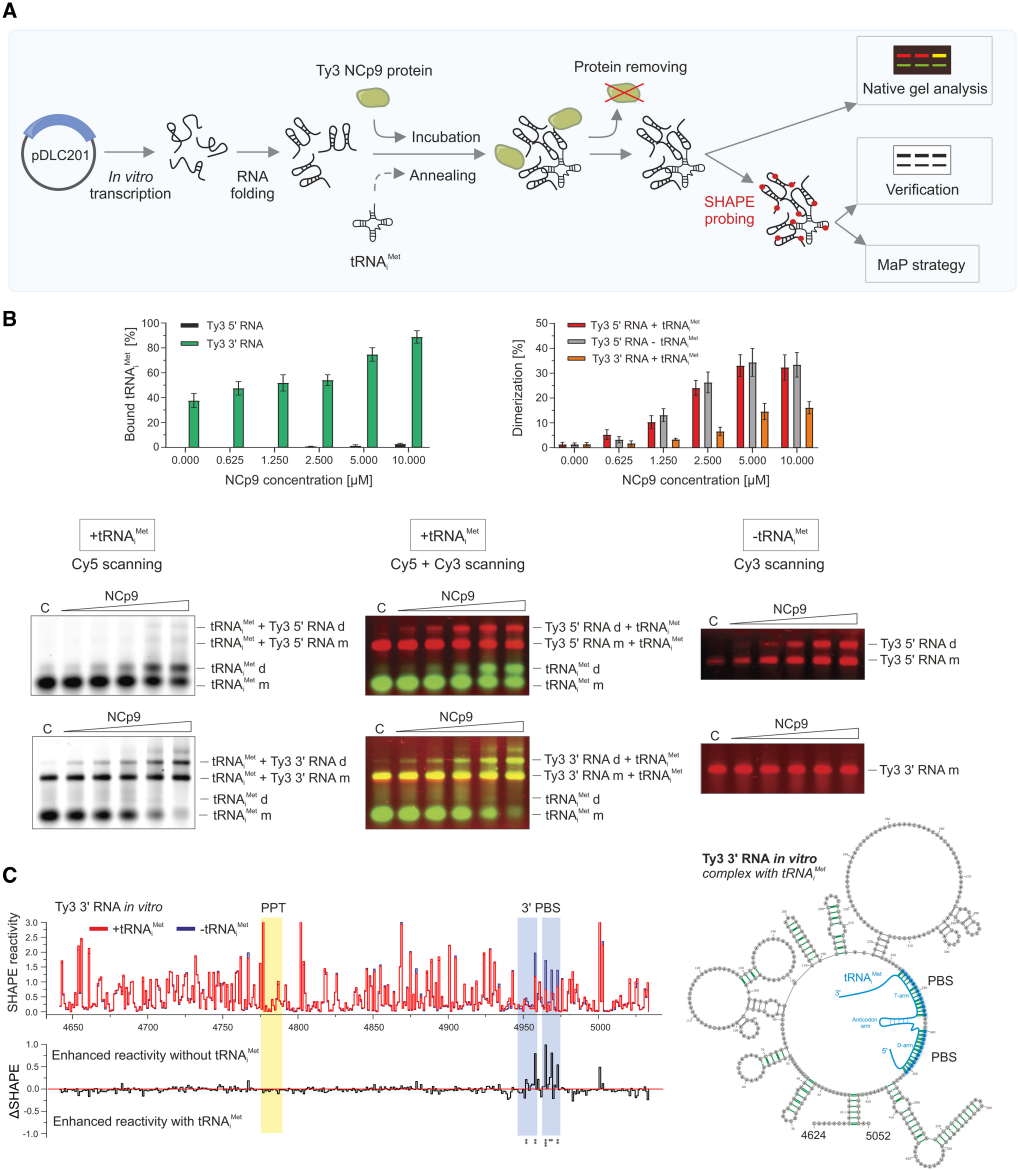
Analysis of NCp9-induced RNA–RNA interactions. (**A**)> Schematic presentation of the applied strategy. (**B**) NCp9-mediated Ty3 5′–5′ and 3′–3′ RNA dimerization and tRNA_i_^Met^ annealing assays. The graphs represent the percentage of bound tRNA_i_^Met^ (left) and dimerized Ty3 RNA (right) with increasing concentrations of NCp9. Representative agarose gels for experiments with the addition or without tRNA_i_^Met^ are presented below. Ty3 RNAs were labeled with Cy3, tRNA_i_^Met^ with Cy5. Lanes C represent protein-free control reactions. Cy3 scan of gels are shown in Supplementary Figure S5. (**C**) The step plot (top) and difference plot (bottom) of NAI reactivities for Ty3 3′ RNA in the presence and absence of tRNA_i_^Met^. *Cis*-acting sequences are marked with colored boxes. Nucleotide positions with statistically significant differential reactivity were marked by asterisks (SHAPE reactivity drop in tRNA_i_^Met^ presence >0.15 and a *P*-value <0.05, using the Student's *t*-test). The MFE model for the Ty3 3′ RNA *in vitro* with the tRNA_i_^Met^ annealed is presented on the right. HP bps are marked in green.

Efficient retroelement replication relies on intermolecular RNA–RNA interactions, such as forming a gRNA dimer or a gRNA-tRNA complex. To better comprehend these processes for Ty3, we utilized a simplified *in vitro* system analogous to the one employed for characterizing these interactions in Ty1 (28,45). We prepared two short transcripts covering the 5′ or 3′ part of the mini Ty3 element—5′ RNA: +1–429 nt and 3′ RNA: +4624–5052 nt. We then incubated fluorescently labeled transcripts and tRNA_i_^Met^ (Cy3 and Cy5, respectively) with increasing amounts of NCp9 protein, and RNA complex formation was assayed by gel electrophoresis (Figure 4A). Consistent with prior study (17), we observed efficient tRNA_i_^Met^ annealing to the 3′ RNA (∼80% of bound tRNA) and to a significantly lesser extent to 5′ RNA (∼3% of bound tRNA) (Figure 4B, Supplementary Figure S5). It may be caused by the shorter sequence complementary to tRNA_i_^Met^ than in the 3′ RNA (8 versus 24 nt) and its lower GC content (Table 1). It results in a stronger folding energy of 3′ PBS-tRNA_i_^Met^ (summed parts 1 and 2) than 5′ PBS-tRNA_i_^Met^. Moreover, the folding energy of 3′ PBS-tRNA_i_^Met^ is comparable to tRNA_i_^Met^. We also detected specific tRNA_i_^Met^ dimers formed via the interaction of palindromic sequences at their 5′ ends. The tRNA_i_^Met^ dimer co-linked to the 5′ and 3′ PBS sequences in two gRNA strands is thought to maintain the Ty3 gRNA dimeric state (17). Indeed, our *in vitro* assays showed the 3′ RNA dimers and the higher order multimers formed only when tRNA_i_^Met^ was present in the mixture (Figure 4B). Interestingly, in contrast to others, we found a more significant conversion toward the dimeric state for the 5′ RNA (up to 40%), independent of tRNA_i_^Met^ addition. This observation suggested that the Ty3 gRNA can dimerize via direct base-pairing of sequences located within its 5′ end, similar to Ty1 and retroviruses (28,46).

The SHAPE-MaP data for 5′ and 3′ transcripts *in vitro* showed a strong correlation with those obtained for the corresponding regions in the Ty3 gRNA *in vivo* (Supplementary Figure S6). The correlations suggested their similar folding to the full-length gRNA, with only a few differences resulting from the sequence shortening. Further comparative analysis of SHAPE-MaP reactivity profiles for 3′ RNA mapped in the absence or presence of tRNA_i_^Met^ revealed local changes strictly in the 3′ PBS region, proving the exact site of tRNA_i_^Met^ binding (Figure 4C). The highest SHAPE reactivity changes were observed for the second part of 3′ PBS, which agrees with its higher GC bp content and stronger folding energy (Table 1). However, we could not characterize the structural properties of the 5′ RNA bound by tRNA_i_^Met^ due to the low efficacy of this complex formation *in vitro*. In a tRNA-free state, 5′ PBS was unreactive, most probably due to engagement in intramolecular base-pairing with nt + 172–191 (Supplementary Figure S7). Similar to 5′ PBS, this sequence was also protected toward the SHAPE reagent independently of tRNA_i_^Met^ presence or absence in the mixture.

### Identification of a candidate Ty3 gRNA dimerization site

**Figure 5.**
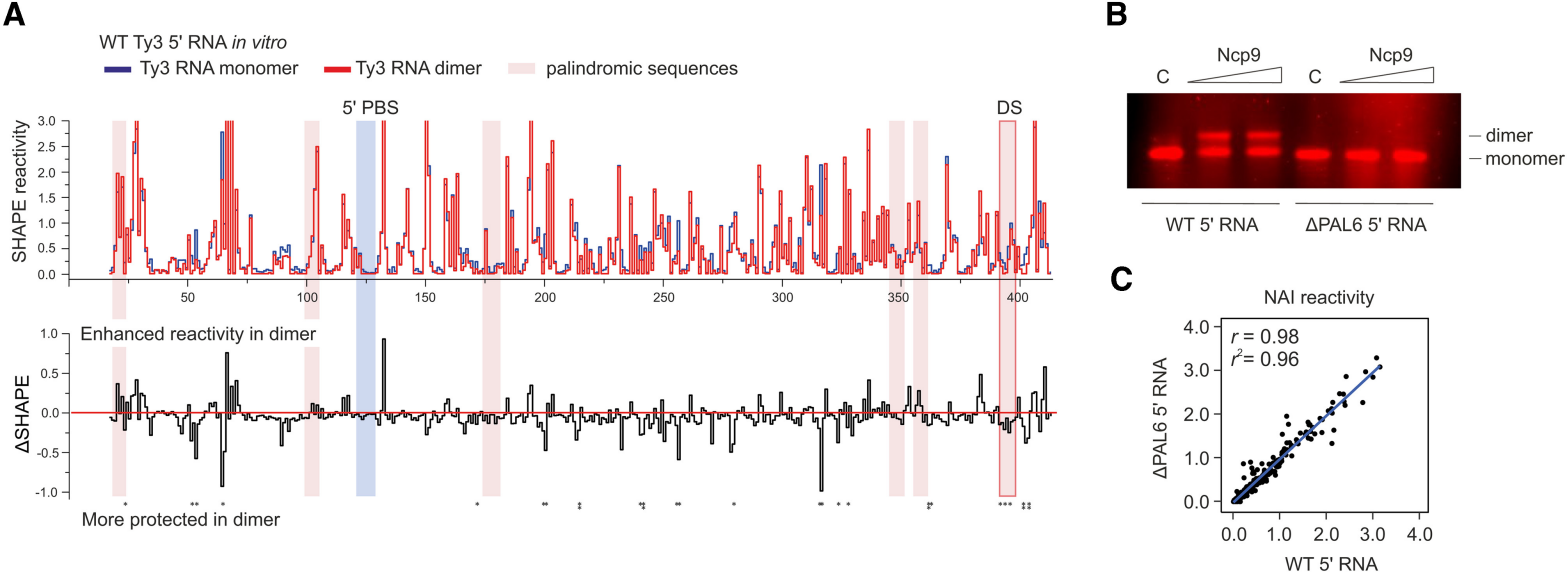
Identification of *cis*-acting sequence important for Ty3 5′ RNA dimerization. (**A**)> The step plot (top) and difference plot (bottom) of NAI reactivities for the Ty3 5′ RNA *in vitro* in the monomeric and NCp9-induced dimeric state. PBS and palindromic sequences (_18_AUUAAU_23_, _99_CUCGAG_104_, _174_UAGCUA_179_, _345_ACAUGU_350_, _355_GAUAUC_360_, _391_CUGCAG_396_) are marked with colored boxes. Nucleotide positions with statistically significant differential reactivity were marked by asterisks (SHAPE reactivity drop in dimer >0.15 and a *P*-value <0.05, using the Student's *t*-test). (**B**) Protein-mediated dimerization assay for WT and ΔPAL6 5′ RNAs; concentrations of NCp9: 5 and 10 μM. Lanes C represent protein-free control reactions. (**C**) Pearson correlation (*r*) plot of normalized SHAPE reactivities between WT and ΔPAL6 5′ RNAs *in vitro* datasets.

To identify the dimerization site, we initially searched for the palindromic sequences in the 5′ end of Ty3 gRNA. This was based on the assumption that Ty3 could follow a retroviral dimerization paradigm, where two gRNA strands interact via short palindromic sequences from their 5′ ends (46). We identified six palindromic sequences, three in 5′ UTR and three in *GAG* ORF (Figure 5A). To determine palindromes that may be functional, we performed a structural analysis of the NCp9-induced Ty3 5′ RNA monomer and dimer using SHAPE-MaP. Since the maximal efficiency of dimerization was ∼40%, we applied a mathematical data deconvolution that allows one to determine the secondary structures of individual RNAs in a mixture of the conformers (28,29). We detected a very strong correlation between the SHAPE reactivity datasets obtained for monomer and dimer *in vitro* (r = 0.98). However, the in-depth analysis of SHAPE profiles revealed statistically significant reactivity decreases for the palindrome 6 in the dimeric state (Figures 5A and Supplementary Figure S8). Concurrently, we did not find statistically relevant reactivity drops in other palindromic sequences that could support their involvement in the Ty3 5′ RNA dimerization.

These observations prompted us to further verify the involvement of selected palindromes in Ty3 gRNA dimerization. We found that deletion of the palindrome 6 (PAL6) results in complete inhibition of dimerization *in vitro*, even though the ΔPAL6 5′ RNA still contained the other five palindromes (Figure 5B). SHAPE-MaP analysis revealed that the 5′ RNA shortening has no significant impact on its folding (*r* = 0.98, Figure 5C), indicating that dimerization inhibition is not due to the overall 5′ RNA structure rearrangement. The PAL6 (_391_CUGCAG_396_) is located within the Ty3 gRNA lowSS region, further supporting its potential functional importance (Figure 3A, B). Taken together, the GC-rich PAL6 is required to form the Ty3 5′ RNA dimer, and we propose that it might serve as the primary dimerization site (DS) *in vitro*.

### Structural analysis of Ty3 gRNA *cis*-acting elements *in vivo*

**Figure 6.**
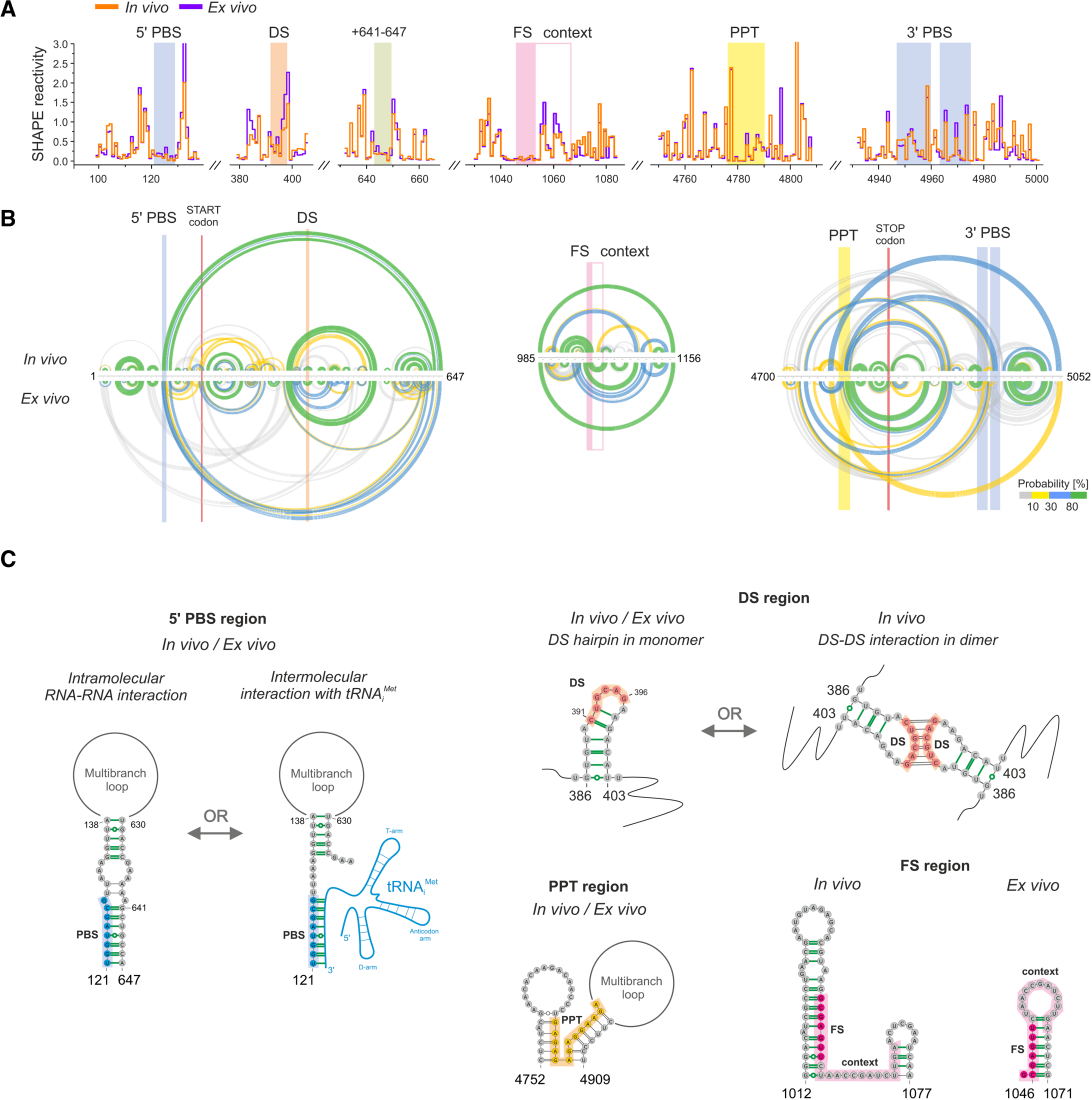
SHAPE-MaP-based analysis of Ty3 gRNA *cis*-acting elements. (**A**)> The step plot of *in vivo* and *ex vivo* SHAPE reactivities for the selected regions of gRNA. *Cis*-acting sequences and 5′ PBS interaction partner are marked with colored boxes. (**B**) Comparative analysis of base-pairing probabilities, represented by arc plots for regions containing *cis*-acting sequences. *Cis*-acting sequences and start or stop codons are marked with colored boxes. (**C**) Structural context of Ty3 gRNA *cis*-acting sequences. *In vivo* HP bps are marked in green.

As a result of the parallel occurrence of Ty3 at different stages of the replication in yeast, the *in vivo* SHAPE data represented the average score from the nucleus, cytoplasm, retrosomes, and VLPs. Moreover, it is unclear how much of the *in vivo* Ty3 gRNA pool is in dimerized or tRNA_i_^Met^-bound form. In total yeast RNA probed *ex vivo*, tRNA_i_^Met^ is still present and can bind to Ty3 3′ RNA spontaneously even without the support of proteins (Figure 4B). In Ty3 gRNA, the 5′ PBS exhibited a low level of SHAPE reactivity *in vivo* and *ex vivo*, resembling the SHAPE pattern obtained for *in vitro* probed Ty3 5′ RNA (Figures 6A and Supplementary Figure S7). However, it was challenging to dissect whether the 5′ PBS is occupied by tRNA_i_^Met^ or intramolecularly constrained in Ty3 gRNA. SHAPE–based structure predictions suggested highly probable base-pairing of PBS with nt + 641–647 (Figure 6B, C). Indeed, nt + 641–647 were mainly unreactive *in vivo* and *ex vivo* (Figure 6A). This interaction cannot occur in the 5′ RNA (+1–429), where an alternative 5′ PBS base-pairing with nt + 172–191 is created (Supplementary Figure S6). Nevertheless, these data showed that apart from tRNA_i_^Met^ binding, the 5′ PBS has a high tendency for intramolecular base-pairing. In contrast, the 3′ PBS segments of Ty3 gRNA were mainly reactive *in vivo* and *ex vivo* (Figure 6A). It suggests their weaker propensity to internal base-pairing but does not exclude tRNA_i_^Met^ binding to the 3′ end of gRNA. It is highly probable that the unbound Ty3 gRNA fraction dominates over that with tRNA_i_^Met^ annealed and camouflages the effect of intermolecular tRNA_i_^Met^-Ty3 gRNA interaction in SHAPE reactivity profiles. We found evidence to support our hypothesis through *in vitro* experiments on Ty3 3′ RNA (Figure 4C). As a result of very efficient tRNA_i_^Met^ binding, we observed a strong SHAPE reactivity drop in the 3′ PBS but not complete protection. This is because some unbound Ty3 3′ RNA was still present in the mixture.

In contrast to the tRNA_i_^Met^-Ty3 gRNA complex, the reconstitution of the Ty3 gRNA dimers in the protein-free *ex vivo* conditions is highly unlikely. These dimers are heat-sensitive (13) and thus disrupted during the applied RNA isolation procedure. Moreover, Ty3 protein is needed for efficient Ty3 RNA dimerization (Figure 4B). For Ty3 gRNA *in vivo*, we detected statistically significant drops in SHAPE reactivity for the newly identified DS/PAL6 sequence, compared to the *ex vivo* state (Figures 6A and Supplementary Figure S9). This effect was not observed for other palindromic sequences. This result additionally supports DS involvement in Ty3 gRNA dimerization. The DS is located *in vivo* in the apical part of the highly probable short hairpin (Figure 6B, C), which suggests that the kissing-loop dimer is first formed, similar to the dimerization model proposed for retroviruses (46,47).

Like in other retroelements, Ty3 *POL* ORF undergoes translation as a result of +1 frameshifting (FS). The Ty3 FS event is mediated by a 7-nt sequence (_1046_GCGAGUU_1052_) and stimulated by the low availability of the tRNA specific for the hungry serine codon (AGU) (43). Additionally, a 12-nt stimulatory ‘context’ sequence nearby prolongs translational pause and increases the frameshifting about 7.5-fold (48). The exact mechanism underlying the stimulation in Ty3 frameshifting remains unknown, but it was postulated that the primary sequence of the ‘context’, not its secondary structure, stimulates frameshifting. We found the ‘context’ sequence highly reactive and mainly unpaired in both gRNA states. Inversely, the FS sequence remained unreactive toward SHAPE reagent both *in vivo* and *ex vivo* and is localized in the stem of the highly probable hairpin (Figure 6A–C).

We also found that the polypurine tract (PPT, _4776_GAGAGAGAGGAAGA_4789_), which serves as a primer for Ty3 plus-strand synthesis, is located in a base-paired region in both gRNA states (Figure 6A–C). However, these pairings were predicted with lower probability, and boundary nucleotides exhibited high or moderate SHAPE modification levels. Studies indicate that the 5′ and 3′ ends of PPT contribute to RNase H recognition and are more sensitive to substitutions than internal positions, which may suggest the need for their higher accessibility (49,50).

### Ty3 gRNA possesses potential pseudoknots and G-quadruplex motifs

**Figure 7.**
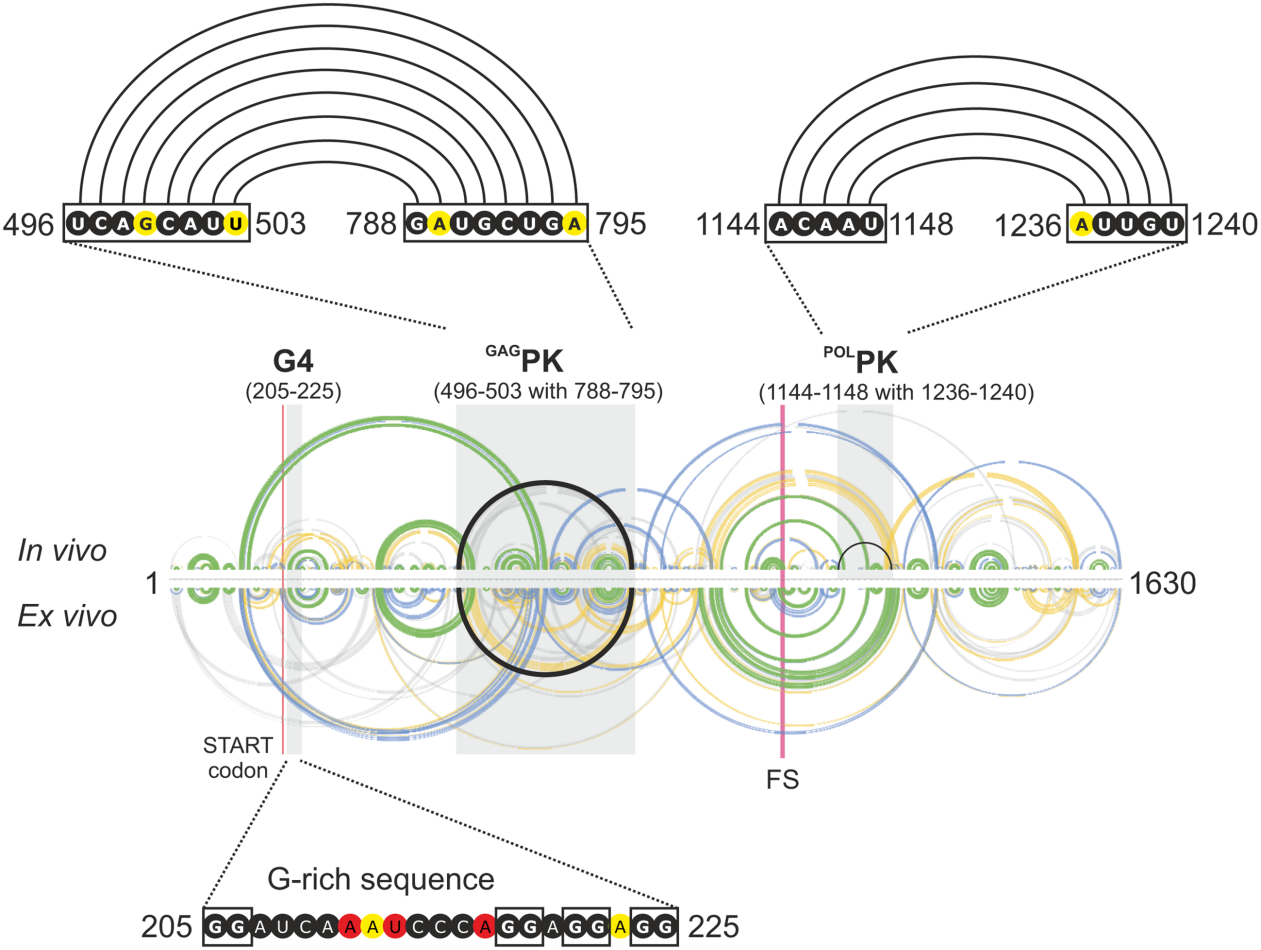
Potential pseudoknots and G-quadruplex predicted in Ty3 gRNA. The ^GAG^PK and ^POL^PK interactions are presented as black arcs. Black frames indicate nts involved in PK or G-quadruplexes, with their SHAPE reactivity. Base-pairing probabilities and SHAPE reactivities are colored with respect to scale in Figure 3.

Many RNA viruses utilize pseudoknot structures to control replication, translation, and the switching between these processes (51–53). Ty1 and HIV-1 gRNA contain experimentally validated pseudoknots, which are functionally important, as their disruption results in an inhibition of replication (45,54–56). Also, RNA G-quadruplexes regulate the life cycle of multiple viruses, including HIV-1 and SARS-CoV-2, by modulating the efficiency of reverse transcription or translation (57,58). *De novo* prediction and experimental validation of G-quadruplexes and pseudoknots, especially *in vivo*, is challenging. MFE structure algorithms are not able to identify such RNA motifs. Therefore, to identify *de novo* potential RNA pseudoknots in Ty3 gRNA, we used the SHAPE-MaP data as constraints for the dedicated ShapeKnots software (33). This analysis revealed two potential pseudoknots *in vivo* – in the *GAG* ORF (^GAG^PK) and the *POL* ORF (^POL^PK) in the proximity of the FS (Figure 7). In contrast to ^POL^PK, the ^GAG^PK was also predicted to be formed *ex vivo*. The ShapeKnots results were sustained by SHAPE-MaP data since nucleotides engaged in both pseudoknots were mainly unreactive in SHAPE-MaP experiments. In addition, we performed *in silico* predictions of potential RNA G-quadruplexes using QGRS Mapper (34). We found only one potential G-quadruplex in Ty3 gRNA at the very beginning of the *GAG* ORF (12 nt after the AUG codon) (Figure 7). In this case, SHAPE data also supported the computational prediction result since guanosines engaged in potential G-quadruplex motif formation remained unreactive in Ty3 gRNA under both experimental conditions. Nevertheless, these RNA motifs' existence in the cells needs further experimental validation.

### Identification of common structural features of retroelement RNA genomes

We searched for common gRNA secondary structure characteristics across diverse retroelements as there are deep evolutionarily relationships between retroelements (59). For this purpose, we compared the SHAPE data obtained for Ty3 gRNA with those determined for yeast Ty1 retrotransposon and the HIV-1 retrovirus (24,25,36,60). These retroelements share important similarities in genome organization and replication cycle, but the HIV-1 gRNA is almost twice as long (9.2 kb) and encodes additional regulatory proteins and the Env, responsible for infectivity.

**Figure 8.**
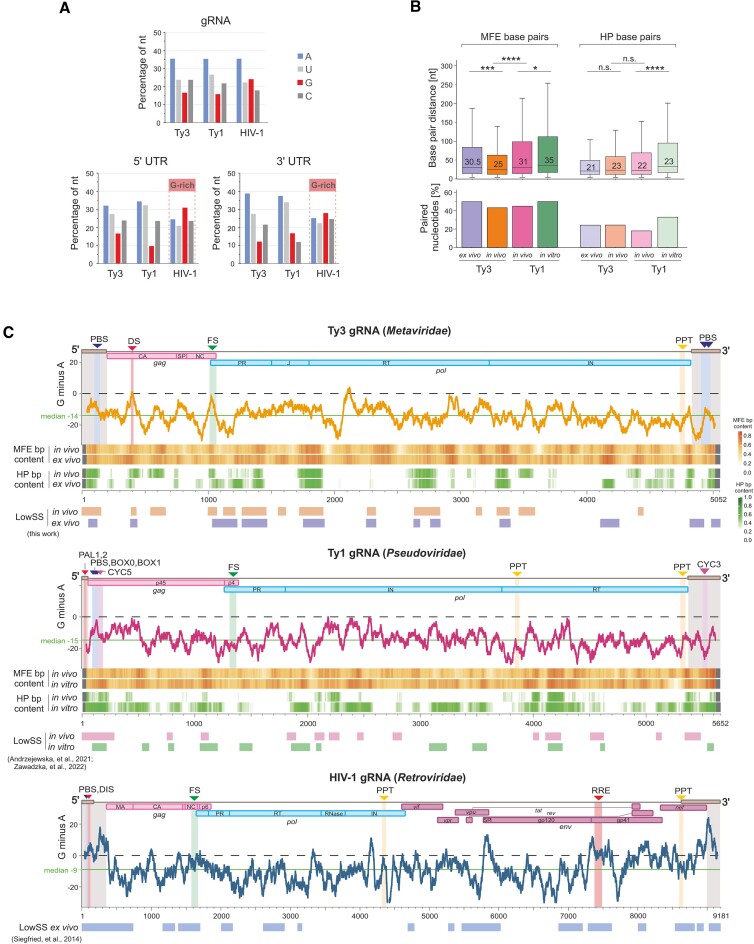
Comparative analysis of Ty3, Ty1, and HIV-1 RNA genomes. (**A**)> Bar plot analysis of nucleotide composition in CDS, 5′ UTR, and 3′ UTR. (**B**) Distribution of MFE and HP bp distances with medians and the percentage of nucleotides engaged in these pairings. (**C**) Profiles of G minus A comparison for gRNA sequences. The number of As was subtracted from the number of Gs (G minus A) in a 75-nt sliding window. The green solid line indicates the median, and the black dashed line indicates where the difference between the number of Gs and As is zero. Values above the dashed line indicate a higher G concentration than A. Heatmaps representing Ty3 or Ty1 bp content (MFE and HP) are shown below the G minus A profiles. The lowSS regions are shown on the bottom. Their locations for Ty3 gRNA were taken from this work, for Ty1 gRNA from (24,25); and for HIV-1 gRNA from (36). PBS, BOX0, BOX1 – primer binding sites, DS, PAL1, PAL2, and DIS – dimerization sequences, CYC5, CYC3 – cyclization sequences, FS – frameshift sequence, PPT – polypurine tract, RRE – Rev response element.

The gRNA of Ty3 is highly enriched in adenosines (∼36%), similar to Ty1 and HIV-1 (Figure 8A). The overall base composition of the viral genome was shown to be related to local RNA structure stability (61,62). A-rich structures may facilitate RNA unfolding and refolding, resulting in less stable secondary structures prone to the formation of alternative conformers, while regions rich in Gs may stabilize functionally critical structures. In HIV-1 gRNA, accumulation of Gs was found in the 5′ and 3′ UTRs and the functional *cis*-acting sequences across the coding sequence—FS and the Rev response element (RRE) (Figure 8A, C). In contrast to HIV-1, we observed guanosine accumulation neither in 5′ nor in 3′ UTRs of both Ty elements (Figure 8A). However, we found Ty3 FS and DS are enriched in Gs, even though there is a lack of G domination over A (Figure 8C). This analysis indicates that the Ty3 retrotransposon, as evolutionarily more related to HIV-1, displays a better correlation between the sequence composition and the location of *cis*-elements, in contrast to Ty1, where this correlation was very low.

SHAPE-directed structure models of Ty1 and Ty3 gRNA *in vivo* were characterized by a lower number and distance of MFE pairings, compared to cell-free state (Figure 8B). The more detailed heatmap of the local MFE bps content showed that highly structured regions are dispersed across these genomes, and their location is extensively shared between experimental states (Figure 8C). Interestingly, the analysis focused on *in vivo* HP bps revealed that the Ty3 gRNA possesses more regions of HP bps accumulation than Ty1 gRNA (Figure 8B, C). Simultaneously, we found higher similarity in HP bps content, distance, and distribution profiles between the Ty3 *in vivo* and *ex vivo* states, compared to *in vivo* state and *in vitro* transcribed Ty1 gRNAs (Figure 8B, C). A common characteristic for the *in vivo* state of both Ty elements was a high accumulation of HP bps within the RT domain.

Furthermore, our studies showed that stable and highly structured (lowSS) regions constitute up to 30% of Ty1 and Ty3 gRNAs (Figure 8C) (24,25). For HIV-1 gRNA, identification of lowSS regions was performed only for cell-free state and they encompass about 40% of the sequence (36) (Figure 8C). More recently, a high structural heterogeneity across the entire HIV-1 gRNA was also shown in cells using the DREEM algorithm (60). Altogether, these sequence and structural comparisons demonstrate that gRNAs from diverse retroelements possess a mosaic secondary structure with regions of higher structural stability interspersed with dynamic ones. Importantly, Ty1, Ty3, and HIV-1 have well-characterized and functionally analogous *cis*-acting sequences in their gRNAs. The common feature is a localization of sequences involved in tRNA annealing, gRNA dimerization, cyclization and packaging in the well-structured 5′ end of gRNA (Figure 8C). In contrast, the FS is located in a structurally stable region of gRNA for Ty3 and HIV-1 but not for Ty1.

We also looked for the correlation between the location of retrotransposon protein domain boundaries and the local level of gRNA structure, as it was previously clearly observed for HIV-1 (63). For Ty3, we observed an evident increase in RNA structure level in the region linking the J and RT domains in *POL* ORF, and a little increase at the beginning of the CA in *GAG* ORF (Supplementary Figure S10). Thus, the correlation is much less evident than observed for HIV-1. For Ty1 retrotransposon, most protein domain boundaries do not exhibit a higher level of RNA structure, excluding the correlation between RNA structure and protein folding.

## Discussion

The yeast Ty3 LTR-retrotransposon is an informative model system for studying retrotransposition mechanisms and the impact of transposable elements on host genomes (5). In this work, we present the complete structural map for the yeast Ty3 retrotransposon RNA genome using the SHAPE-MaP strategy both in living cells and *ex vivo*. A comparative analysis of Ty3 gRNA structure in different experimental states facilitates a more correct interpretation of the relationship between RNA structure, function, and cellular factors. Only several studies provide such comparisons for viral RNAs (24,64–69). Consistent with these studies, we observed a slight shift in Ty3 gRNA structure towards a more unfolded state *in vivo*. Nevertheless, over half of the predicted MFE bps and three-fourths of the HP bps are shared between the *in vivo* and *ex vivo* Ty3 gRNA states. This structural similarity indicates that many interactions are independent of the folding environment Ty3 gRNA encounters *in vivo*. Like other single-stranded RNA viruses, Ty3 gRNA has a complex secondary structure enriched in stable, specific structural motifs. We identified a well-folded core formed by more stable regions without the support of cellular factors, and it encompasses about 20% of the Ty3 gRNA sequence. Among identified structurally stable regions with low SHAPE reactivity and Shannon entropy (lowSS regions), half is included in the well-folded core, but the others are specific for each experimental state. The stable motifs were shown to be functionally necessary for the replication of RNA viruses (36,67,70–72). Accordingly, Ty3 lowSS regions encompass *cis*-acting sequences, like 5′ PBS, FS, and DS, but the role of other gRNA stable motifs remains to be explored.

Analogous to other retroelements, Ty3 gRNA is present in the dimeric form in VLP (13). The sequences involved in Ty3 gRNA dimerization were not clearly identified, and it remains unknown whether, like in retroviruses, dimerization is essential for Ty3 RNA packaging into VLP. An early study suggests that the Ty3 gRNA dimer *in vitro* is maintained by the interaction between two tRNA_i_^Met^ molecules co-linked to the 5′ and 3′ PBS sequences (17). However, further study demonstrates that deletion of 5′ and 3′ PBS does not prevent Ty3 gRNA packaging (20). Therefore, either tRNA_i_^Met^ is unnecessary for Ty3 gRNA dimerization or dimerization is not a pre-requisite for this RNA packaging. Alternatively, different mechanisms of dimerization are also possible. We showed that the transcript encompassing Ty3 gRNA 5′ end dimerizes efficiently without tRNA_i_^Met^ mediation. This finding indicates that Ty3 retrotransposon follows the common mechanism of retroelement gRNA dimerization, which occurs through the direct interaction of *cis*-acting palindromic sequences from the 5′ end. And importantly, we propose a novel functional Ty3 RNA sequence – DS/PAL6, that directly drives the dimerization process. We also showed that DS/PAL6 is less reactive *in vivo* than *ex vivo*, suggesting its involvement in Ty3 gRNA dimerization in yeast. Further studies are needed to explain the role of dimerization via DS/PAL6 for Ty3 gRNA packaging into VLPs.

The primary function of tRNA_i_^Met^ in Ty3 replication is the initiation of gRNA reverse transcription, and its annealing to both 5′ and 3′ PBS sequences is necessary for this process (17). Since SHAPE does not discriminate intra- and intermolecular RNA interactions, resolving the issue of tRNA_i_^Met^ annealing to 5′ PBS is challenging due to its low reactivity in all experimental states and tendency to intramolecular interactions. Nevertheless, SHAPE results obtained for 3′ PBS suggest that only a tiny fraction of Ty3 gRNA interacts with tRNA_i_^Met^*in vivo* and *ex vivo*.

Studies for other RNA viruses highlight the role of -1 FS structural context in modulating frameshifting efficiency (53). In contrast, the +1 frameshift mechanism in Ty3 is postulated to rely mainly on the specific peptidyl-tRNA availability but can be additionally induced by the ‘context’ sequence (48). Our results provide a structural basis to further understand how the stable secondary structure of the Ty3 FS element along with the single-stranded ‘context’ sequence regulate frameshifting. However, further experimental validation is required to confirm this hypothesis, such as studying the frameshift rate when a well-folded hairpin with FS sequence is disrupted, or some stable motifs are incorporated within the ‘context’ sequence.

In the cytoplasm, Ty3 gRNA co-localizes with Ty3 proteins in P-body-associated foci (termed retrosomes), where VLP assembly occurs (9,16). Apart from the UTRs, the *POL* region plays an independent role in the localization of Ty3 gRNA in the cytoplasmic foci (20). A level of ribosome occupancy of *POL* was shown to be inversely correlated with Ty3 gRNA localization in cytoplasmic foci. It was proposed that ribosomes may impede access to located in *POL* sequences important for binding host factors involved in Ty3 localization. However, these sequences are not distinguished explicitly so far. We identified several stable lowSS regions across Ty3 *POL*, which represent attractive candidates for further Ty3 gRNA localization studies.

To date, Ty3 is the first endogenous retroelement in the *Metaviridae* family, along with the yeast Ty1 retrotransposon in the *Pseudoviridae* family, whose gRNA structure has been determined in a native *in vivo* state (24,25). RNA genomes of both Ty retrotransposons are significantly enriched in A nucleobases and exhibit some general structural similarities, including a mosaic RNA structure, which is more open *in vivo* than under cell-free conditions. Nevertheless, due to higher HP bp content, Ty3 gRNA structural motifs may be more stable *in vivo* than Ty1 gRNA. These HP bps, together with the same RNA posttranscriptional processing, may contribute to better structural similarity between Ty3 gRNA *in vivo* and *ex vivo* states. Moreover, only Ty3 gRNA is enriched for G nucleobases in functionally essential sequences, similar to the HIV-1 gRNA (61). However, there is no evident correlation between RNA structure level and protein domain boundaries as was found in HIV-1 (63).

Overall, the comprehensive characterization of Ty3 gRNA secondary structure, along with a comparative analysis with diverse retroelements, broadens our understanding of the relationship between gRNA architecture and the replication process. This study also provides the basis for understanding the function of retrotransposon RNA transactions at a molecular level.

## Supplementary Material

gkae494_Supplemental_File

## Data Availability

Raw sequencing data can be obtained from https://www.ncbi.nlm.nih.gov/bioproject/ and can be accessed under accession code PRJNA1072546. All further data generated or analyzed during this study are included in this published article and its supplementary file.
