# Supplementary material for: Mapping the structural landscape of the yeast Ty3 retrotransposon RNA genome
Source: Nucleic Acids Res. 2024 Jun 12;52(16):9821–37. doi: 10.1093/nar/gkae494 (PMC11381356; doi:10.1093/nar/gkae494)
Supplement: gkae494_Supplemental_File [file gkae494_supplemental_file.pdf]

SUPPLEMENTARY TABLES AND FIGURES

Mapping the structural landscape of the yeast Ty3 retrotransposon RNA genome

Table S1. Primers used in the study.

PRIMER	SEQUENCE (5'-3')
**Colony PCR**	
PF_Gal_Ty3_part	GCTTCTAATCCGTACTTCAATATAG
PR_Ty3_part	CGTCTTTTAATGTCTCTGGTTTG
PF_AMP_part	CCTATCTCAGCGATCTGTCTAT
PR_AMP_part	GAGTATTCAACATTTCCGTGTCG
**Reverse transcription and PCR**	
PF_amp1	TAAGTAACATTCCGTG
PR_amp1	CGTCTTTTAATGTCTC
PF_amp2	GACCGAAAAAGCTGCCATAAT
PR_amp2	CTAAAGTTATATGCAGGTCATTGAT
PF_amp3	TCAAATACGAAATCTACGAGACC
PR_amp3	GCCAGGTTTAATTTCAATATCATG
PF_amp4	ACAACAGAAGTATAGAGAGATCATAC
PR_amp4	TAGTGGAGCTATTTTCTGGATTC
PF_amp5	CATTGGAAACATTTAGACACGG
PR_amp5	CATCTGCGACAACGTTCTTG
PF_amp6	TATCATTACAAAACAAGAACGAACC
PR_amp6	GTTTTCCTTGTAGCTATGAAGTG
PF_amp7	TATCAATGGATTTTGTGACAGGATT
PR_amp7	GAAATGGTCCGACGTATATTTG
PF_amp8	TTATTGTTAAACATAGGAGATCACG
PR_amp8	GTCAAAACAGTTTATCAGATTAATTCAC
PF_invitro_5end	TAAGTAACATTCCGTG
PR_invitro_5end_16	GCCCACTGAGCAGCGG
PR_invitro_5end_12	GCCCACTGAGCA
PR_invitro_5end_ΔPAL6	TACACAATACGACTGGCATCATT
PF_invitro_3end	CTACACACAAAACTTACT
PR_invitro_3end	GTCAAAACAGTTTATCAG
**PCR for *in vitro* transcription templates**	
PF_5'_Ty3	TAATACGACTCACTATAGTAAGTAACATTCCGT
PR_5'_Ty3	GCCCACTGAGCAGCGGGGTT
PR_5'_Ty3ΔPAL6	TACACAATACGACTGGCATCATT
PF_3'_Ty3	TAATACGACTCACTATAGAGGGGCCAGC
PR_3'_Ty3	GTCAAAACAGTTTATCAGATTAATTCACGGAATG

Supplementary Figure Legends

Figure S1. Pearson correlation (*r*) plots of normalized SHAPE reactivities between different NAI concentrations and modification times *in vivo* and *ex vivo* Tests were performed for the first amplicon.

Figure S2. Box plot analysis of mutation rates for each nucleotide identity for modified and untreated samples from Ty3 gRNA probing under *in vivo* and *ex vivo* conditions. The boxes represent the interquartile range; a line indicates the median. Significance was computed by the Wilcoxon rank-sum test; ****$P < 0.0001$.

Figure S3. Identification of significant SHAPE reactivity differences between *in vivo* and *ex vivo* Ty3 gRNA states using the ΔSHAPE framework. To identify local and global differences, SHAPE profiles were smoothed with 3-nt and 51-nt sliding windows, respectively. Regions that meet the standard score and Z-factor criteria are marked with green and violet shadings.

Figure S4. Thermal stability of NCp9-induced Ty3 5'-5' and 3'-3' RNA dimers after protein removal. For 3' RNA, $tRNA_i^{Met}$ was present in the reaction. Ty3 RNAs were labeled with Cy3, $tRNA_i^{Met}$ with Cy5.

Figure S5. Cy3 scan (signal for Ty3 5' or 3' RNA) of representative agarose gels for NCp9-mediated 5'-5' and 3'-3' Ty3 RNA dimerization assays in the presence of $tRNA_i^{Met}$. Lanes marked C represent protein-free samples.

Figure S6. Base-pairing probabilities for Ty3 5' RNA and Ty3 3' RNAs *in vitro* compared to Ty3 gRNA *in vivo. Cis*-acting sequences and start or stop codons are marked with colored boxes. Pearson correlation (*r*) plot of normalized SHAPE reactivities between *in vivo* and *in vitro* datasets.

Figure S7. The step plot of NAI reactivities for Ty3 5' RNA *in vitro* in the presence and absence of $tRNA_i^{Met}$. PBS and predicted interaction partner are marked with colored boxes. The arc plot of this interaction is presented below.

Figure S8. Identification of significant SHAPE reactivity differences between monomeric and dimeric states of Ty3 5' RNA *in vitro* using the ΔSHAPE framework. Before ΔSHAPE calculation, SHAPE profiles were smoothed with a 3-nt sliding window. Regions that meet the standard score and Z-factor criteria are marked with green and violet shadings. PBS and palindromic sequences are marked with colored boxes.

Figure S9. The step plot (top) and difference plot (bottom) of NAI reactivities for the 5' end of Ty3 gRNA *in vivo* and *ex vivo*. Nucleotide positions that exhibit statistically significant differential reactivity (SHAPE reactivity drop *in vivo* > 0.15 and a *P*-value < 0.05, using the

Student's t-test) were marked by asterisks according to statistical significance. PBS and palindromic sequences are marked with colored boxes.

Figure S10. Comparative analysis of the gRNA structure level (median SHAPE and Gini index profiles) and the protein domain boundaries for Ty3 and Ty1 genomes.

Figure S1.

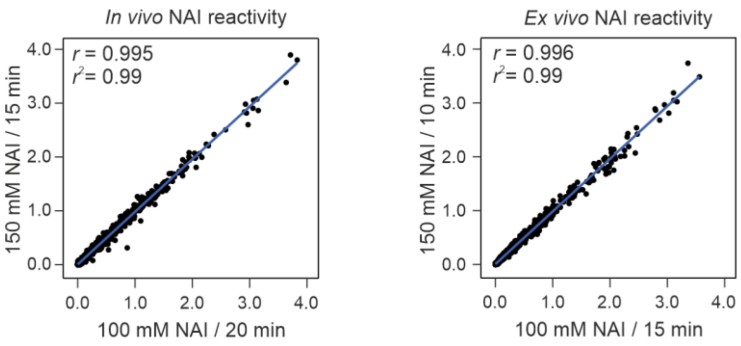

Figure S2.

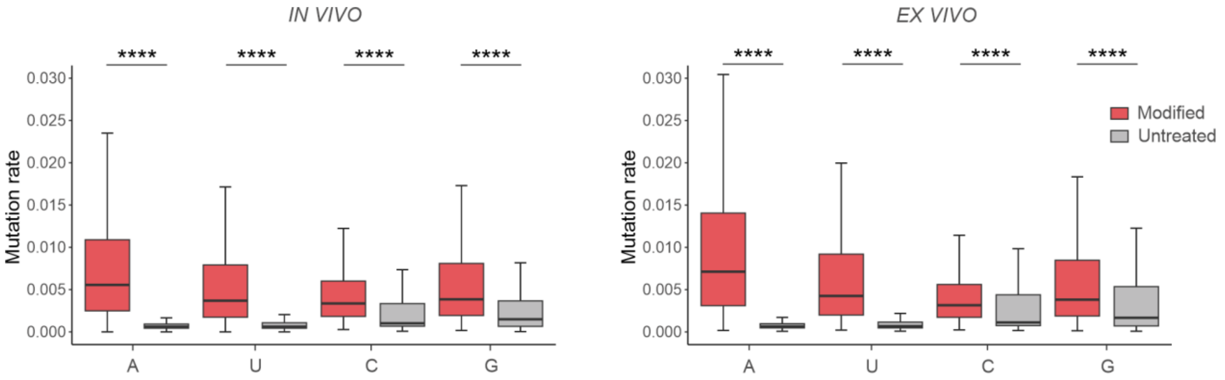

Figure S3.

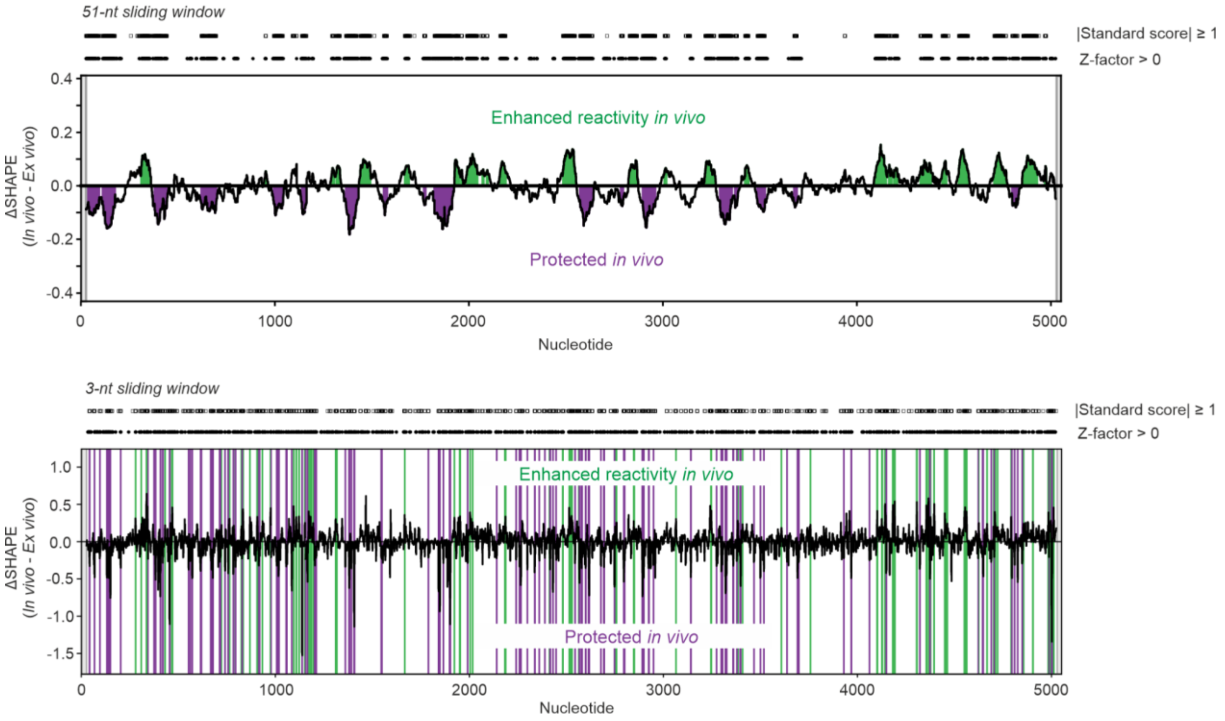

Figure S4.

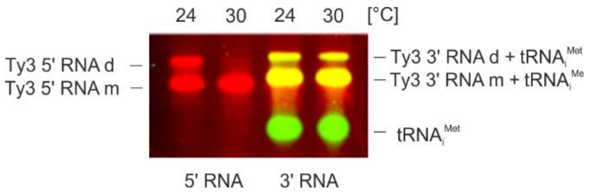

Figure S5.

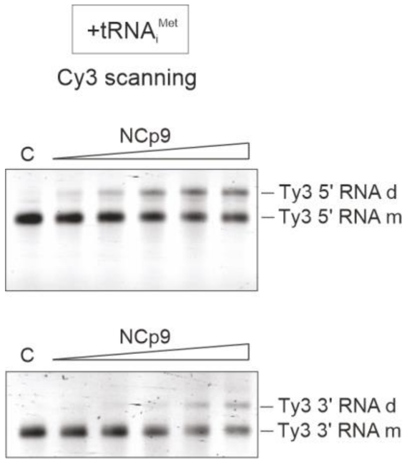

Figure S6.

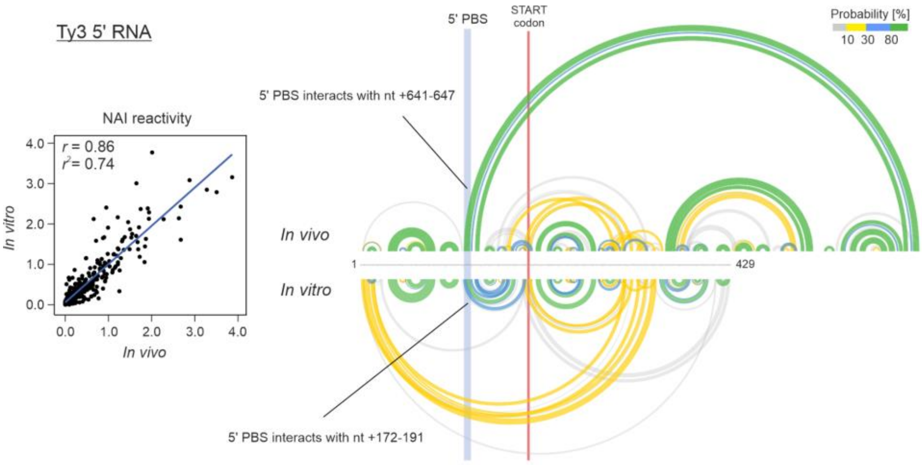

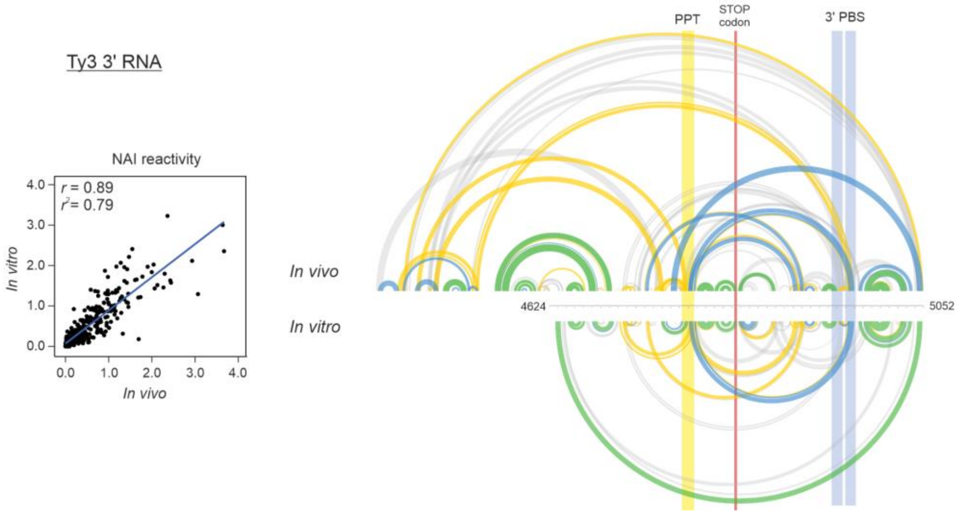

Figure S7.

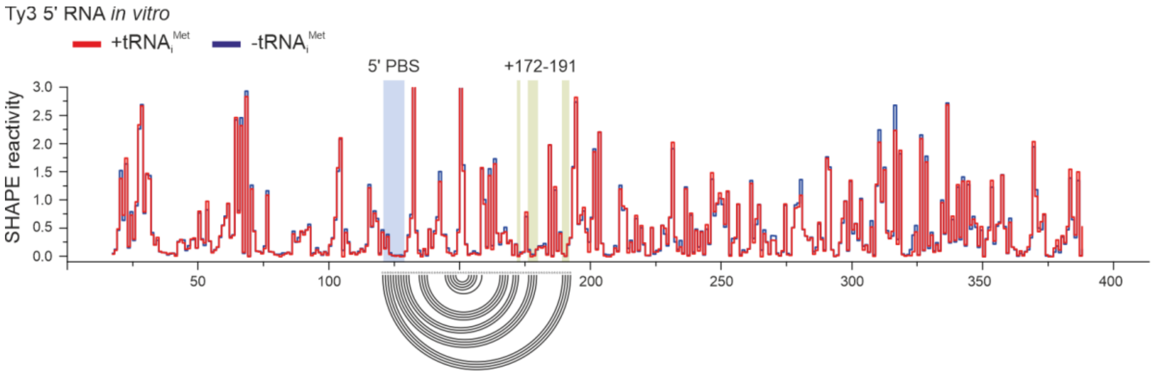

Figure S8.

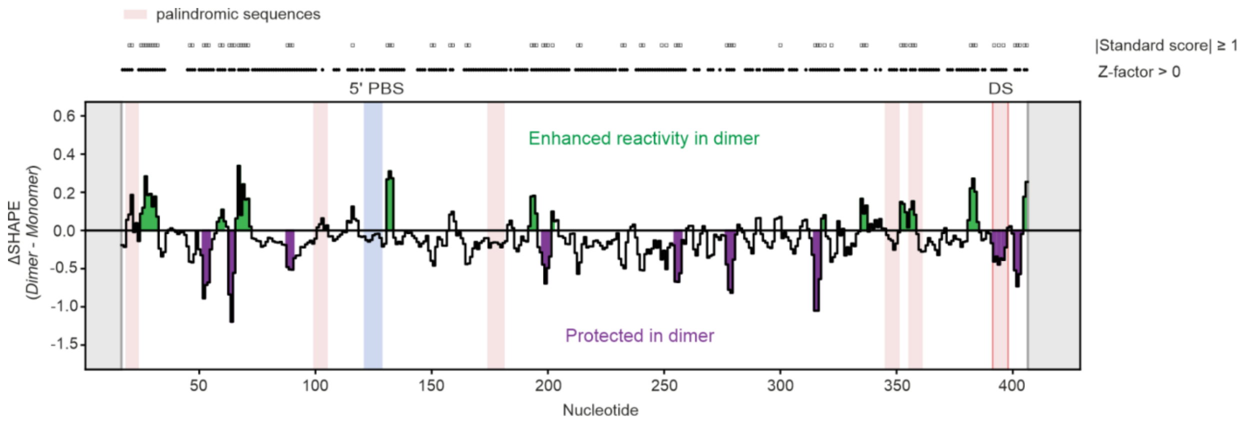

Figure S9.

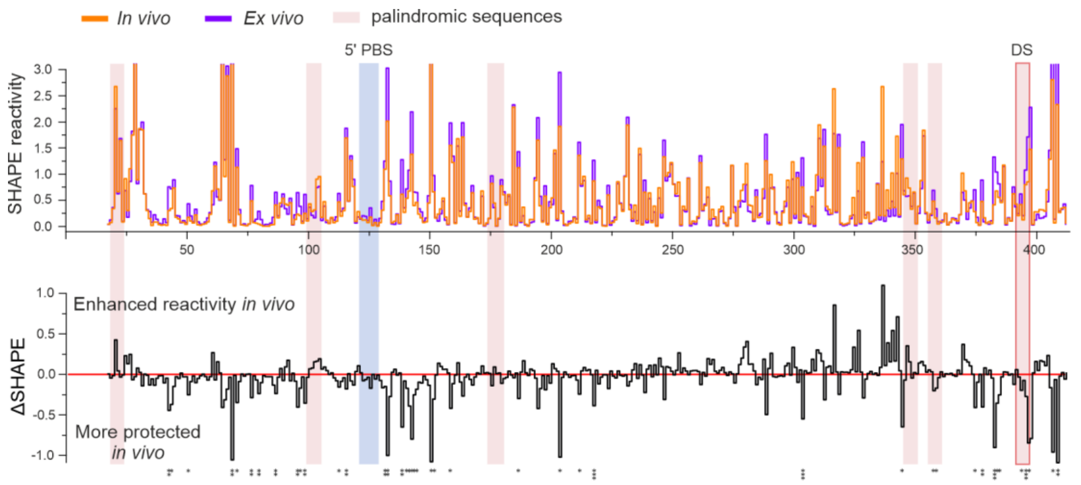

Figure S10.

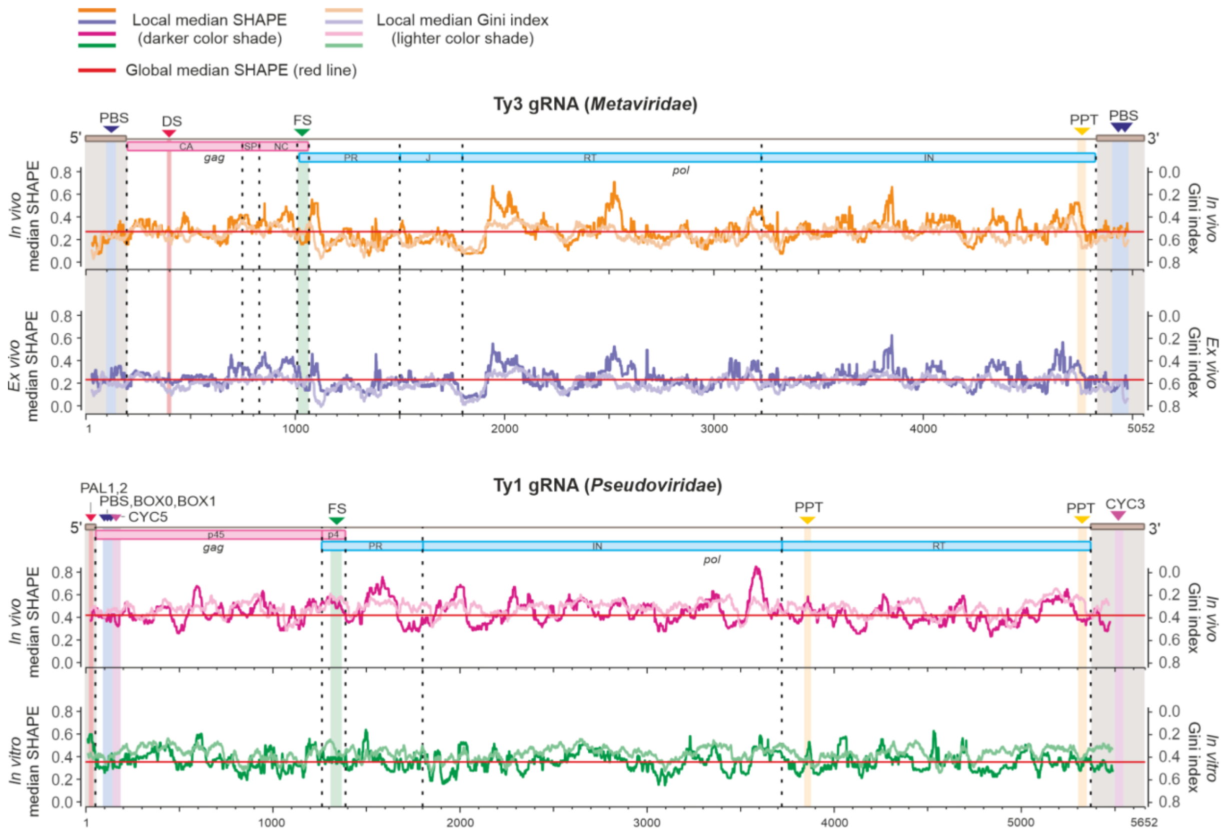

Local median SHAPE (darker color shade)
Local median Gini index (lighter color shade)
Global median SHAPE (red line)
Ty3 gRNA (Metaviridae)
PBS
DS
FS
PPT
PBS
5'
3'
CA
SP
NC
gag
PR
J
RT
IN
pol
In vivo median SHAPE
In vivo Gini index
Ex vivo median SHAPE
Ex vivo Gini index
1
1000
2000
3000
4000
5052
Ty1 gRNA (Pseudoviridae)
PAL1,2
PBS,BOX0,BOX1
CYC5
FS
PPT
PPT
CYC3
p45
p4
gag
PR
IN
RT
pol
In vivo median SHAPE
In vivo Gini index
In vitro median SHAPE
In vitro Gini index
1
1000
2000
3000
4000
5000
5652
